# Post-translational modifications of hnRNP A1 differentially modulate retroviral IRES-mediated translation initiation

**DOI:** 10.1093/nar/gkaa765

**Published:** 2020-09-22

**Authors:** Aldo Barrera, Hade Ramos, Jorge Vera-Otarola, Leandro Fernández-García, Jenniffer Angulo, Valeria Olguín, Karla Pino, Andrew J Mouland, Marcelo López-Lastra

**Affiliations:** Laboratorio de Virología Molecular, Instituto Milenio de Inmunología e Inmunoterapia, Departamento de Enfermedades Infecciosas e Inmunología Pediátrica, Escuela de Medicina, Pontificia Universidad Católica de Chile, Marcoleta 391, Santiago, Chile; Laboratorio de Virología Molecular, Instituto Milenio de Inmunología e Inmunoterapia, Departamento de Enfermedades Infecciosas e Inmunología Pediátrica, Escuela de Medicina, Pontificia Universidad Católica de Chile, Marcoleta 391, Santiago, Chile; Laboratorio de Virología Molecular, Instituto Milenio de Inmunología e Inmunoterapia, Departamento de Enfermedades Infecciosas e Inmunología Pediátrica, Escuela de Medicina, Pontificia Universidad Católica de Chile, Marcoleta 391, Santiago, Chile; Laboratorio de Virología Molecular, Instituto Milenio de Inmunología e Inmunoterapia, Departamento de Enfermedades Infecciosas e Inmunología Pediátrica, Escuela de Medicina, Pontificia Universidad Católica de Chile, Marcoleta 391, Santiago, Chile; Laboratorio de Virología Molecular, Instituto Milenio de Inmunología e Inmunoterapia, Departamento de Enfermedades Infecciosas e Inmunología Pediátrica, Escuela de Medicina, Pontificia Universidad Católica de Chile, Marcoleta 391, Santiago, Chile; Laboratorio de Virología Molecular, Instituto Milenio de Inmunología e Inmunoterapia, Departamento de Enfermedades Infecciosas e Inmunología Pediátrica, Escuela de Medicina, Pontificia Universidad Católica de Chile, Marcoleta 391, Santiago, Chile; Laboratorio de Virología Molecular, Instituto Milenio de Inmunología e Inmunoterapia, Departamento de Enfermedades Infecciosas e Inmunología Pediátrica, Escuela de Medicina, Pontificia Universidad Católica de Chile, Marcoleta 391, Santiago, Chile; HIV-1 RNA Trafficking Laboratory, Lady Davis Institute at the Jewish General Hospital, Montréal, Québec H3T 1E2, Canada; Department of Medicine, McGill University, Montréal, Québec H4A 3J1, Canada; Laboratorio de Virología Molecular, Instituto Milenio de Inmunología e Inmunoterapia, Departamento de Enfermedades Infecciosas e Inmunología Pediátrica, Escuela de Medicina, Pontificia Universidad Católica de Chile, Marcoleta 391, Santiago, Chile

## Abstract

The full-length mRNAs of the human immunodeficiency virus type-1 (HIV-1), the human T-cell lymphotropic virus type-1 (HTLV-1), and the mouse mammary tumor virus (MMTV) harbor IRESs. The activity of the retroviral-IRESs requires IRES-transacting factors (ITAFs), being hnRNP A1, a known ITAF for the HIV-1 IRES. In this study, we show that hnRNP A1 is also an ITAF for the HTLV-1 and MMTV IRESs. The MMTV IRES proved to be more responsive to hnRNP A1 than either the HTLV-1 or the HIV-1 IRESs. The impact of post-translational modifications of hnRNP A1 on HIV-1, HTLV-1 and MMTV IRES activity was also assessed. Results show that the HIV-1 and HTLV-1 IRESs were equally responsive to hnRNP A1 and its phosphorylation mutants S4A/S6A, S4D/S6D and S199A/D. However, the S4D/S6D mutant stimulated the activity from the MMTV-IRES to levels significantly higher than the wild type hnRNP A1. PRMT5-induced symmetrical di-methylation of arginine residues of hnRNP A1 enabled the ITAF to stimulate the HIV-1 and HTLV-1 IRESs while reducing the stimulatory ability of the ITAF over the MMTV IRES. We conclude that retroviral IRES activity is not only dependent on the recruited ITAFs but also relies on how these proteins are modified at the post-translational level.

## INTRODUCTION

The group-specific antigen (Gag), is the major structural component of retroviral particles and is the viral protein responsible for orchestrating the late stages of the retroviral replication cycle (1). Translation of the full-length, or genomic, retroviral mRNA is the only source of Gag (Pr55) and Gag-Pol (Pr160) precursor polyproteins. During HIV-1 virion maturation, Gag is cleaved into the structural proteins matrix (MA, p17), capsid (CA, p24), the nucleocapsid (NC, p7), and p6 and the spacer peptides (p1 and p2) (2,3). In addition to the structural proteins, cleavage of the Gag-Pol precursor, the product of a −1 frameshift, delivers the viral enzymes the protease (PR), reverse transcriptase (RT) and integrase (IN) (4). Many retroviruses have evolved alternative strategies designed to exploit the host's protein synthesis machinery to ensure translation initiation of the genomic mRNA to guarantee an adequate expression of the Gag polyproteins (5,6).

Retroviral transcripts possess a triphosphate-linked 7-methylguanosine (m7-GTP) cap structure at their 5′ end and a poly(A) tail at their 3′ extremity and can initiate translation following a canonical cap-dependent mechanism (5,6). Also, ample evidence shows that translation initiation of the full-length mRNA of some retroviruses can occur via an internal ribosome entry site (IRES) in addition to canonical scanning (5,6). Examples are the full-length mRNAs of the human immunodeficiency virus type 1 (HIV-1) (7–10), the etiological agent of AIDS, the human T-cell lymphotropic virus type 1 (HTLV-1) (11,12), that causes adult T cell leukemia and HTLV-associated myelopathy/tropical spastic paraparesis, and the mouse mammary tumor virus (MMTV) (13), associated with breast tumors in mice and probably in humans (14,15).

Translation initiation of the HIV-1 full-length mRNA has been extensively studied (16), becoming the prototypical retroviral IRES (5,6). However, little is known about IRES-mediated translational initiation in other retroviruses, in particular how these IRESs are controlled by host cell factors (5,6). The full-length HIV-1 mRNA harbors two IRESs, one within the 5′untranslated region (5′UTR) of the viral transcript (HIV-1 IRES) (7,9,10), and the focus of this study, while the other lies within the Gag protein-coding region (HIV-1 Gag-IRES) (8,17). The HIV-1 IRES activity enables the synthesis of Gag at stages of the viral cycle when cap-dependent translation initiation is inhibited (7,18–20). During its replication, HIV-1 suppresses cap-dependent translation initiation by blocking the cell cycle in G2/M (21,22), by inducing oxidative and osmotic stress (20,23), and by viral protease-mediated eIF4G and PABP cleavage (24–27). Despite cap-dependent inhibition induced by these events, Gag synthesis is sustained throughout the viral replication cycle (16). The HIV-1 IRES also allows full-length mRNA translation in the presence of picornavirus proteases, which cleave eIF4G and hinder cap-dependent translation initiation (18,19), and when the mammalian target of rapamycin (mTOR) activity is pharmacologically inhibited (28). Interestingly, when cap-dependent translation is inhibited either by the picornavirus proteases or by the inhibition of mTOR, synthesis of the HIV-1 Gag is abruptly downregulated but not shutdown (18,19,28). The synthesis of Gag protein is then partially restored, suggesting that a switch from canonical to non-canonical translation initiation occurs (18,19,28).

The molecular mechanisms governing the switches from cap- to IRES-dependent translation initiation of the full-length HIV-1 mRNA remain unknown (18,19,28). Furthermore, how the HIV-1 IRES, or any other retroviral IRES, recruits the translational machinery also continues unclear (5,6,16). However, compelling results suggest that the activity of the HIV-1 IRES, and other retroviral IRESs, rely on host factors such as the eukaryotic initiation factors (eIF) 4A and eIF5A (29–31), ribosomal protein S25 (12,29,32), and the availability of IRES *trans-acting factors* (ITAFs) that activate or repress its activity (9,23,29,30,32–35). For the HIV-1 IRES, one such ITAF is the heterogeneous nuclear ribonucleoprotein A1 (hnRNP A1) (10,23,36), a host protein that binds the viral genomic mRNA in the nucleus and is exported to the cytoplasm as part of the full-length HIV-1 mRNA ribonucleoprotein (RNP) complex (23). In the cytoplasm, hnRNP A1 enhances HIV-1 IRES activity (10,23,36). The role played by hnRNP A1 as an ITAF is not limited to the HIV-1 IRES, as the protein also modulates the IRESs of other viral and cellular mRNAs (37–48). How hnRNP A1 activates or inhibits IRES activity is still incompletely understood. However, post-translational modifications (PTMs) of hnRNP A1 impact on the activity of cellular IRESs. For example, protein arginine methyltransferases (PRMT)-induced methylation of hnRNP A1 enables the protein to stimulate Cyclin D1 (CCDN1), cellular Myelocytomatosis (c-MYC), hypoxia-inducible factor-1α (HIF1α), estrogen receptor α (ESR1), and cyclin-dependent kinase inhibitors 1B (CDKN1B) IRESs (41), while they induce a hnRNP A1 associated suppression of the IRES activity from the X-linked inhibitor of apoptosis (XIAP) and B-cell lymphoma extra-large (BCL-XL) mRNAs (49). Akt-induced phosphorylation of hnRNP A1 on its serine residue 199 (S199) attenuates the protein′s ability to stimulate the CCDN1 and c-MYC IRESs (50). The ribosomal S6 kinase 2 (S6K2)-induced phosphorylation on sites S4 and S6 counteract the inhibitory effect exerted by hnRNP A1 over the XIAP and BCL-XL IRESs, thereby increasing IRES activity (42). Thus, the PTMs of hnRNP A1 fine-tune the protein's ability to modulate the IRES-dependent gene expression from its cellular target mRNAs. Interestingly, the mitogen-activated protein kinase (MAPK)-interacting protein kinases (Mnk)-mediated phosphorylation of the c-terminal domain of hnRNP A1 contributes to HIV-1 IRES-mediated translation initiation (23). Treatment with inhibitors of Mnk activity selectively reduced HIV-1 IRES-mediated translation initiation without impacting cap-dependent translation initiation or hnRNP A1 cytoplasmic localization (23). So, as shown for cellular IRESs, it is likely that PTMs of hnRNP A1 also fine-tune retroviral IRES activity.

Because ITAF activity of hnRNP A1 on host cell IRESs can be modulated by PTMs (41,42,49,50), we examined the involvement of hnRNP A1 phosphorylations and protein arginine methyltransferase 5 (PRMT5)-methylations on retroviral IRES-mediated translation initiation. Our results showed that hnRNP A1 PTMs are involved in the differential tuning of the HIV-1, HTLV-1, and MMTV IRES activities. These findings unveil a novel mechanism of translational control of retroviral gene expression ruled by PTM events of ITAFs, such as hnRNP A1. Thus, we provide new evidence showing that retroviral IRES activity not only depends on the trans-acting factors recruited to the 5′UTR of the full-length mRNA but also relies on how these proteins are post-translationally modified, adding a new degree of complexity to the overall understanding of retroviral cap-independent translation initiation.

## MATERIALS AND METHODS

### Plasmids

The dual-luciferase (dl) plasmids dl HIV-1 IRES (7,34), dl HTLV-1 IRES (12), dl MMTV IRES (13) have been previously described. The HIV-1 pNL4.3-RLuc provirus vector was kindly provided by Dr R. Soto-Rifo (Programa de Virología, ICBM, Universidad de Chile) (51). The pCMV3-hnRNP A1-HA was purchased from Sino Biological Inc. (#HG16537-CY; Wayne, PA, USA). The sequence of all constructs used in this study was verified (Psomagen Inc., Rockville, MD, USA).

### Site-directed mutagenesis

Point mutants of the pCMV3-hnRNP A1-HA plasmid were generated using the Thermo Fisher Scientific Phusion Site-Directed Mutagenesis Kit (#F-541, Thermo Fisher Scientific Inc. Life Technologies Inc., Carlsbad, CA, USA) and primers described in Table 1, designed according to the manufacturer's protocol. The polymerase chain reaction (PCR) assays were performed in a Veriti ™ 96-well Thermal Cycler (#4375768, Thermo Fisher Scientific Inc.). The screening of all transformants was performed by sequencing (Psomagen Inc., Rockville, MD, USA).

**Table 1.** Primers used to generate the hnRNP A1 point mutants

Sequence (5′P→3′)	Substitution	Domain	Reference
F: CCATGTCTAAGGCAGAGGCTCCTAAAGAG	S4A, S6A	N-terminus	(42)
R: TACCAAGCTTGGTGGCGG			
F: CCATGTCTAAGGACGAGGATCCTAAAGAGCCC	S4D, S6D		
F: TCGAAGTGGTGCTGGAAACTTT	S199A	RRM2/Gly-rich	(40)
R: CCTCTTTGGCTGGATGAAGCACTA			
F: TCGAAGTGGTGATGGAAACTTT	199D		
F: TTCAGTGGTAAGGGTGGCTTTG	R218K, R225K	Gly-rich	(41)
R: GTTTCCTCCCTTACCGAAGTTG			

### Cell culture and drug treatments

Human embryonic kidney cells (HEK 293T, ATCC, CRL-11268) were grown in Dulbecco modified Eagle's medium (DMEM; #SH30022, HyClone, GE Healthcare Life Sciences, Logan, UT, USA) containing 10% fetal bovine serum (#SH30910, Hyclone, GE Healthcare Life Sciences), 1% penicillin–streptomycin (1000 U/ ml) (#SV30010, Hyclone, GE Healthcare Life Sciences), and 1% amphotericin B (25 mg/ml) (#SV30078.01, Hyclone, GE Healthcare Life Sciences), at 37°C in a 5% CO_2_ atmosphere. The PRMT5 inhibitor EPZ015666 (#SML1421, Sigma-Aldrich, St. Louis, MO, USA) was resuspended in DMSO. HEK 293T were seeded at 1×10^5^ or 5×10^4^ cells per well in a 24- or 48-well culture plates, respectively. Twenty-four hours later, cells at 60% confluence were treated with DMSO (control) or EPZ015666 at the indicated compound concentrations for 48 h.

### DNA transfection

HEK 293T cells were seeded at 1.2×10^5^ cells per well in 24-well culture plates. DNA transfection experiments were performed at 70–80% confluency using polyethyleneimine (PEI; GIBCO, Thermo Fisher Scientific Inc, Waltham, MA, USA). The cells were co-transfected with 300 ng of dl-plasmids, together with the indicated concentration (ng) of hnRNP A1-HA plasmids or plasmid pSP64 Poly(A) (#P1241, Promega Corporation); the last was used as a filler DNA to keep the final DNA concentration constant in all transfection assays. In all experiments 24 or 48 h after transfection, the culture medium was removed and the cells were harvested using Passive Lysis buffer supplied with the Dual-Luciferase® Reporter Assay System (#E1960, Promega Corporation, Madison, WI, USA) according to manufacturer's protocols.

### siRNA-DNA co-transfection

HEK 293T cells were seeded at 1×10^5^ cells per well in 24-well culture plates. Endogenous hnRNP A1 protein silencing was performed over 60% confluent cells using the Lipofectamine 2000 system (#11668019, Invitrogen, Thermo Fisher Scientific Inc.). For hnRNP A1 silencing, a silencer RNA (siRNA) duplex (5′-AATGGGGAACGCTCACGGACT-3′; Integrated DNA Technologies, IDT, Coralville, IA, USA) previously described (23), targeting the open reading frame (ORF) of the hnRNP A1 mRNA was used at 150 nM for 48h, together with 200 ng of dl HIV-1 IRES plasmid, or at 100, 150 or 250 nM for 48 hr in combination with 200 ng of pNL4.3-RLuc plasmids. For RLuc silencing, 100 nM of a duplex siRNA targeting the RLuc ORF (Renilla siRNA 2, 5′-UAUAAGAACCAUUACCAGAUUUGCCUG-3′, Integrated DNA Technologies) (12,29,32), was co-transfected with 150 ng of dual-luc plasmids and pSP64 Poly(A) or hnRNP A1-HA plasmids for 48h. The Silencer Select Negative Control #1 siRNA (#4390844, Ambion, Thermo Fisher Scientific Inc.), was used as a non-related scrambled RNA (scRNA) negative control.

### Luciferase assays

The activity of Firefly luciferase (FLuc) and *Renilla* luciferase (RLuc) were measured using the DLR^®^ Assay System (#E1960, Promega Corporation) according to the manufacturer's instructions, on 20 μl of cell lysates using a Sirius Single Tube Luminometer (Berthold Detection Systems GmbH, Pforzheim, Germany). Data are expressed as a percentage of the Relative Luciferase Activity (RLA) or as Relative Translation Activity (RTA); the latter corresponding to the FLuc/RLuc ratio, an index of the IRES activity (7,9,12,13,29,32).

### RNA extraction and real-time RT-qPCR

Cytoplasmic RNA from HEK 293T cells was extracted using the protocol described in (52). Cells were washed twice with PBS 1X (#SH30256, Hyclone, GE Healthcare Life Sciences), incubated with RLNa buffer (10 mM Tris–HCl pH 8, 10 mM NaCl, 3 mM MgCl_2_, 0,5% NP-40, 1 mM DTT) containing 10 U of Ribolock RNase inhibitor (#EO0381, Thermo Fisher Scientific Inc.). After incubation on ice 500 μl of TRIzol reagent (#15596018, Life Technologies Corporation, Thermo Fisher Scientific Inc.) was added to the supernatant, and the RNA was recovered (52). Cytoplasmic RNA was resuspended in 20 μl of nuclease-free water, DNase-treated (#AM1907, Ambion, Thermo Fisher Scientific Inc.), and recovered according to the manufacturer's instructions. The RNA concentration was quantified by nano-spectrophotometry (N60-Implen Nanophotometer, Westlake Village, CA, USA). The real-time RT-qPCR experiments were carried out using the Brilliant II SYBR Green RT-qPCR one Step Master Mix (#600835, Agilent Technologies, Santa Clara, CA, USA). Gag-RLuc-HA RNA was amplified using primers Renilla sense (5′-AGGTGAAGTTCGTCGTCCAACATTATC-3′) and Renilla antisense (5′-GAAACTTCTTGGCACCTTCAACAATAGC-3′) as previously described (29). No-RT-qPCR reactions were carried out to control for contaminant DNA. Glyceraldehyde 3-phosphate dehydrogenase (GAPDH) mRNA was detected with the primers GAPDH sense (5′-TCCACCACCCTGTTGCTGTAG-3′) and GAPDH antisense (5′-ACCCACTCCTCCACCTTTGAC-3′) as previously described (53). Data analysis was performed by the ΔΔCt method previously described (54,55).

### Cytotoxic assay

The cell viability assay was performed using the CellTiter 96^®^ Aqueous One Solution Cell Proliferation Assay (#G358A, Promega Corporation) according to the manufacturer's instruction. Briefly, HEK 293T cells were seeded at 1.5×10^3^ cells per well in a 96-well plate and transfected with the indicated concentrations of sc/siRNAs or treated with DMSO or EPZ015666, or transfected with the indicated amounts of hnRNP A1-HA. 48 h later, the CellTiter 96^®^ Aqueous One Solution Cell Proliferation Assay was added, incubated at 37°C for 2 h, and the absorbance was measured at 495 nm in a Biochrom EZ Read 400 microplate reader (Biochrom, Holliston, MA, USA).

### Surface sensing of translation (SUnSET)

The effect over the global cellular protein synthesis was analyzed using the SUnSET technique, as previously described (56). Briefly, HEK 293T cells were seeded at 1×10^5^ cells per well and treated with DMSO or the indicated concentrations of EPZ015666. Forty-eight hours later, cells were treated with a 10 min pulse of puromycin (1 μg/ml). As a control for translational inhibition, cells were treated with dithiothreitol (DTT, 2.5 mM) for 90 min before the puromycin pulse. *De novo* synthesized proteins containing puromycin were detected by western blot, as indicated below.

### Western blots

Cells were lysed using the Passive Lysis 5X Buffer (#E1941, Promega Corporation), and the concentration of total protein was determined by the Bradford assay using the Bio-Rad Protein Assay (#5000006, Bio-Rad Laboratories, Inc., Hercules, CA, USA). Between 20 and 40 μg of total protein were resolved in a 12% Glycine SDS-PAGE gel and transferred onto a 0.45 μm nitrocellulose membrane (#10600002; GE Healthcare Bio-Sciences 100 Results Way, Marlborough, MA, USA). In the case of the anti-SDMA western blot (41,57,58), a 12% Tricine SDS-PAGE gel and a 0.45 μm polyvinylidene membrane (#88518, Thermo Fisher Scientific Inc.) were used. Membranes were blocked with TBS containing 5% skimmed milk and 0.1% Tween-20 for 1 h at room temperature and incubated overnight at 4°C with the primary antibody. Primary antibodies mouse anti-hnRNP A1 (#SC-32301; Santa Cruz Biotechnology Inc, Dallas, TX, USA) at a 1:5000 dilution, mouse anti-HA (#H9658, Sigma-Aldrich, 3050 Spruce Street, St. Louis, MO, USA) at a 1:5000 dilution, mouse anti-puromycin (#MABE343, EMD Millipore, Temecula, CA, USA) at a 1:15 000 dilution, mouse anti-PRMT5 (#SC-376937, Santa Cruz Biotechnology Inc, Dallas, TX, USA) at a 1:1000 dilution, rabbit anti-SDMA (#13222S, Cell Signaling Technology, Inc., Danvers, MA, USA; kindly shared by Dr F. Valiente-Echeverria, Programa de Virología, ICBM, Universidad de Chile) at a 1:1000 dilution, rabbit anti-p17 HIV-1 (#4811, NIH AIDS Reference and Reagents program; kindly shared by Dr R. Soto-Rifo, Programa de Virología, ICBM, Universidad de Santiago) at a 1:1000 dilution, mouse anti-GAPDH (#MA5-15738; Thermo Fisher Scientific Inc.) at a 1:5000 dilution and mouse anti-Actin (#69100; MP Biomedicals, Santa Ana, CA, USA) at a 1:5000 dilution (loading control) were used. Either a goat anti-mouse or Goat anti-rabbit IgG-horseradish peroxidase (HRP) conjugate (#AP308P, #AP132P; Merck, Darmstadt, Germany) secondary antibodies, both at 1:10 000 dilution were used. Western blots were visualized by enhanced luminescence by a chemiluminescence reaction using 4-hydroxycinnamic acid (#800237, Merck) and luminol (#09253, Sigma-Aldrich) or the SuperSignal™ West Femto Maximum Sensitivity Substrate (#34096, Thermo Fisher Scientific Inc.). For semiquantitative comparative analysis of protein amounts, western blot films (Fuji medical X-ray film Super HR-U 30) were digitalized using a CanonScan 9950F scanner as in (59,60), or membrane chemiluminescence was captured by an Alliance 2.7 imaging system (UVItec Cambridge, Topac Inc., Cohasset, MA, USA). Optical densities (OD) for each band were determined using the ImageJ 1.38 software (Windows version of NIH Image, http://rsb.info.nih.gov/nih‐image/). When indicated, the value (%) corresponds to the ratio (OD value hnRNP A1/OD value GAPDH) expressed relative to the control sample set to 100%.

### HnRNP A1-HA cellular distribution

For cytoplasmic and nuclear compartment experiments, different amounts of hnRNP A1-HA plasmid were transfected in 293T cells for 24 h, then 1.8 ×10^7^ of transfected cells were collected by centrifugation, washed with PBS and resuspended in 1.5 ml of PBS. 500 μl (equivalent to $\sim$6 ×10^6^ cells) were centrifuged at 1000 g for 5 min and pellet was resuspended in 500 μl of RIPA buffer (Tris–HCl pH 7.5 10 mM, EDTA 1 mM, NaCl 150 mM, NP-40 0.5%, sodium deoxycholate 0.5%, SDS 0.1%, plus protease inhibitors (Roche Diagnostics, Indianapolis, IN, USA)) for whole cell lysate (WCL) extraction. 1000 μl (equivalent to ∼1.2×10^7^ cells) were centrifuged and pellet was resuspended in 500 μl of RLNa buffer (10 mM Tris–HCl pH 8, 10mM NaCl, 3 mM MgCl_2_, 0,5% NP-40, 1 mM DTT) and incubated for 5 min on ice and centrifuged at 13 000 rpm for 2 min. Supernatant harboring cytoplasmic fractions were recovered, and the pellet (nuclear fraction) was resuspended in 500 μl of RIPA buffer. Total protein content was determined by Bradford assay (BIO-RAD Laboratories, Inc., CA, USA) in each extract, and 30 μg of total protein were loaded on a 12% Tris-Tricine SDS-polyacrylamide gel (Tricine-gel). The poly-A-binding protein (PABP) and Poly(ADP-ribose) polymerase-1 (PARP-1) were used as a cytoplasmic and nuclear marker, respectively. Mouse monoclonal antibodies anti-PABP (#SC-32318, Santa Cruz Biotechnology), anti-PARP-1 (#SC-136208, Santa Cruz Biotechnology) in 1:1000 dilution and anti-HA, anti-hnRNP A1 in 1:5000 dilution were used as primary antibodies.

### Immunoprecipitation

For the immunoprecipitation (IP) assays, HEK 293T cells were transfected with the wild type (wt)-hnRNP A1-HA (12.5 μg) or the hnRNP A1-HA R218/225K (12.5 μg) plasmids. Twenty-four hours post-transfection, cells were washed with PBS and resuspended in lysis buffer (NaCl 100 mM, EDTA 2 mM, Tris–HCl 50 mM pH 7.5, NaF 50 mM, sodium orthovanadate 1 mM, Triton X-100 1%, protease inhibitors (Roche Diagnostics). The protein concentration in cellular lysates was determined by Bradford assay (BIO-RAD). For IPs, 1 mg of total protein was used together with protein A/G PLUS-Agarose (#SC-2003, Santa Cruz Biotechnology) loaded with an anti-HA mouse monoclonal antibody (#H9658, Sigma-Aldrich). After 16 h at 4°C, with rotation, the beads were washed with lysis buffer three times before directly mixing beads with tricine loading buffer (2×) and heated (95°C for 5 min). The supernatant was recovered by centrifugation and subjected to western blot assay. About one-third of supernatant was loaded on a tricine-gel to confirm the IP of wt-hnRNP A1-HA or the hnRNP A1-HA R218/225K protein by western blot using mouse an anti-HA antibody. The remaining two-thirds of the supernatant was used to evaluate SDMA using rabbit anti-SDMA (Cell Signaling Technology) (41,57,58). The recombinant protein A/G conjugated with HRP was used as the secondary antibody (Thermo Fisher Scientific).

### UV-CLIP

UV-crosslinking immunoprecipitation (CLIP) was performed as described in (35), using HEK 293T cells transfected with the dl HIV-1 IRES plasmid (7.5 μg). Four hours post-transfection, cells were treated with, or without, 10 μM of EPZ015666 and 48 h later cells were washed, covered with of cold PBS 1× (7 ml/10 cm plate), and UV-irradiated (UV-254 nm, 400 mJ/cm^2^) on ice in a UV chamber (UVP-CL-1000, Upland, CA, USA). Cells were scraped, collected by centrifugation at 4°C, and lysed using RIPA buffer (Tris–HCl pH 7.5 10 mM, EDTA 1 mM, NaCl 150 mM, NP-40 0.5%, sodium deoxycholate 0.5%, SDS 0.1%, plus Roche protease inhibitors), 3 μl Riboblock (Fermentas) and 6 μl DNAseRQ1 (Promega) and treated as detailed in (35). The cell lysate was precleared with BSA pre-blocked protein-A/G-Agarose beads (#SC-2003, Santa Cruz Biotechnology Inc, Dallas, TX, USA) (35). 300 μl of cell lysate was saved as input for RNA and protein extraction. BSA-blocked beads (50% slurry) were used for incubation with the mouse anti-hnRNP A1 antibody or mouse control IgG (#SC-2025, Santa Cruz Biotechnology Inc, Dallas, Texas, USA) antibody. 800 μl of cell lysate (∼1.5 mg of total protein) were used for each IP following RNA extraction, and 500 μl of cell lysate was used for each IP following western blotting as previously described (35). The beads were washed twice with 1 ml of RIPA lysis buffer and three times with High Salt buffer (Tris–HCl pH 7.5 50 mM, NaCl 1 M, EDTA 1 mM, NP-40 1%, sodium deoxycholate 0.5%, SDS 0.1%). Proteins were resolved on a 12% Tricine SDS-PAGE gel and transferred to a polyvinylidene membrane (#88518, Thermo Fisher Scientific Inc.), and western blots were conducted as previously described (35), but using the anti-hnRNP A1 antibody at a 1:1000 dilution and the anti-GAPDH antibody at a 1:5000 dilution. Protein A/G conjugated to horseradish peroxidase (HRP) (Thermo Fisher Scientific Inc.) in a 1:10 000 dilution was used as the secondary antibody. RNA extraction, treatment, and quantification by RT-qPCR were performed as detailed in (35). Data analysis was performed by the ΔΔCt method, as previously described and detailed in (35).

### Sequence and statistical analysis

Graphics and statistical analysis were carried out using the GraphPad 6 software (La Jolla, CA, USA) performing an unpaired two-tailed *t*-test for individual comparison or an ordinary one-way or two-way ANOVA with Brown-Forsythe or an ANOVA Kruskal–Wallis test (as indicated in the text) for group comparison. Primer design and sequence alignments were conducted using Geneious 11 and MEGA 7 software.

## RESULTS

### The heterogeneous nuclear ribonucleoprotein A1 participates in HIV-1 protein synthesis

**Figure 1.**
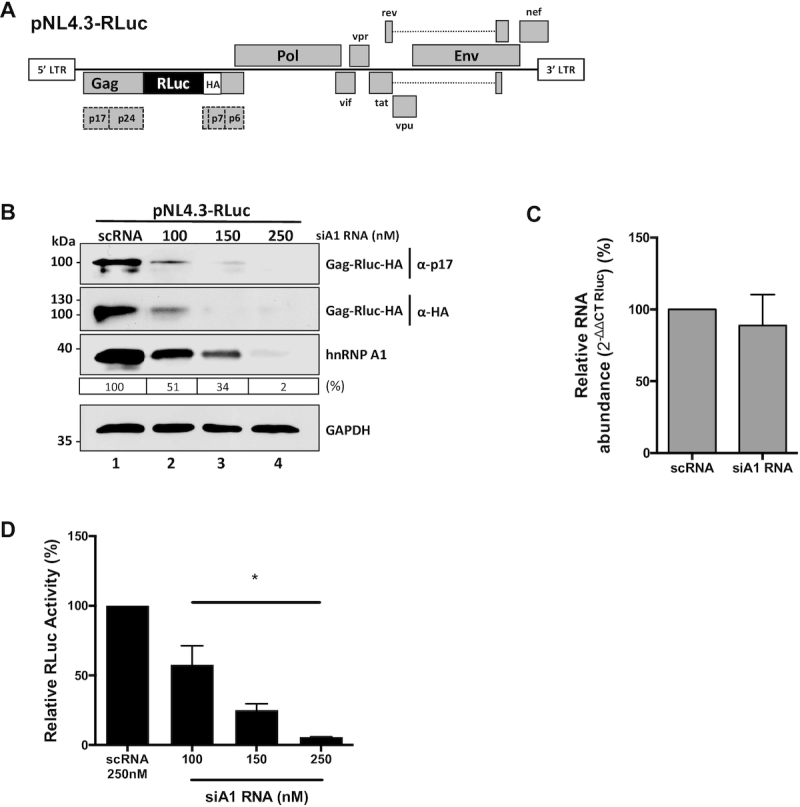
The hnRNP A1 knockdown negatively impacts on Gag protein synthesis from an HIV-1 molecular clone. (**A**) Schematic representation of the complete HIV-1 molecular clone pNL4.3-RLuc described in (51). (**B**) HEK 293T cells were co-transfected with the pNL4.3-RLuc (200 ng) plasmid and increasing concentrations (100, 150 or 250 nM) of a silencer RNA (siRNA) targeting the hnRNP A1 mRNA (siA1), or with a scrambled RNA (scRNA; 250 nM) as a control. The expression of the Gag-Rluc-HA fusion protein and hnRNP A1 was determined 48 h post-transfection by western blot, using the GAPDH protein as a loading control. The relative level of hnRNP A1 protein (%) was estimated based on the intensity of immunoreactive bands by imageJ, as detailed in Materials and Methods. (**C**) Cytoplasmic RNA was extracted from cells expressing pNL4.3-RLuc HIV-1 and treated with the scRNA (250 nM) or with the siA1 RNA (150 nM), and relative RNA levels were determined by real-time RT-qPCR. The RNA abundance was expressed relative to the value obtained for the cells treated with the scRNA set to 100%. Statistical analysis was performed by an unpaired two-tailed *t-test*. (**D**) *Renilla* luciferase activity was measured 48 h post-transfection and is expressed relative to the activity obtained when the pNL4.3-RLuc plasmid was co-transfected with the scRNA. Values represent the mean (±SEM) for three independent experiments, each conducted in duplicate. Statistical analysis was performed by an ordinary one-way ANOVA test (*P<0.05).

HnRNP A1 interacts with the HIV-1 full-length mRNA (61–64), participating in different stages of its metabolism, including splicing (62,65,66), trafficking (23,67), and translation (10,23,36). HnRNP A1 interacts with the HIV-1 5′UTR and promotes its IRES activity (23,64). However, due to conflicting reports regarding the impact of hnRNP A1 on HIV-1 replication (68,69), we decided to assess if hnRNP A1 does play a role in translation of the HIV-1 full-length mRNA. For this, HEK 293T cell lines were transfected with the complete molecular clone pNL4.3-RLuc HIV-1. This molecular clone of HIV-1 is modified in the Gag protein-coding region by the insertion of an hemagglutinin (HA)-tagged Renilla luciferase (RLuc-HA) reporter gene, in frame with the Gag-protein start codon, generating a Gag-RLuc-HA fusion protein (51). This plasmid (Figure 1A), allows a direct evaluation of protein synthesis from the HIV-1 full-length mRNA, using the Gag-RLuc-HA reporter and its luciferase activity as a readout (51). The pNL4.3-RLuc plasmid was transfected in HEK 293T cells together with different concentrations (100–250 nM) of a short interfering RNA (siRNA) that targeted hnRNP A1 mRNA (siA1-RNA) or with a non-related scrambled control siRNA (scRNA; 250 nM). The transient knockdown of hnRNP A1 did not affect cell viability (Supplemental Figure S1A). The impact of the siRNA treatment on the levels of the endogenous hnRNP A1 protein and the Gag-RLuc-HA fusion protein was followed by western blot using GAPDH as a loading control (Figure 1B). The treatment of cells with siA1-RNA reduced the expression of endogenous hnRNP A1, as well as the levels of Gag-RLuc-HA detected either using antibodies against the matrix (p17) region of Gag or against the HA-tag (Figure 1B). Cytoplasmic RNA was extracted from pNL4.3-RLuc HIV-1 transfected cells treated with the scRNA (250 nM) or the siA1RNA (150 nM) and used as a template for quantitative analysis of the pNL4.3RLuc mRNA by an RT-qPCR. No significant changes in transcript levels between treatments were detected, indicating that the decrease in viral protein levels was not associated with a reduction in full-length viral mRNA (Figure 1C). We also determined the luciferase activity of the Gag-RLuc-HA fusion protein. Results showed a significant (*P* <0.05) siA1-RNA concentration-dependent decrease in RLuc activity ($\sim$42–94% reduction) (Figure 1D). These observations confirmed that hnRNP A1 participates in the translation of the HIV-1 full-length mRNA.

### Heterogeneous nuclear ribonucleoprotein A1 modulates HIV-1 IRES-activity

**Figure 2.**
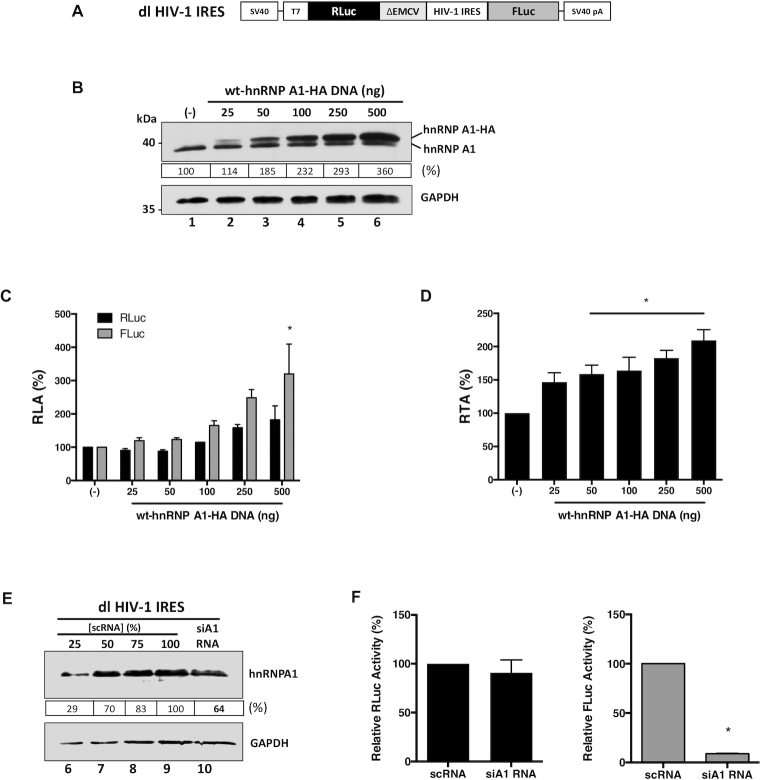
HnRNP A1 is and ITAF for the HIV-1 IRES. (**A**) Schematic representation of dl HIV-1 IRES plasmid described in (7). The *RLuc/FLuc* bicistronic reporter mRNA is expressed from the SV40 promoter and receives a poly(A) tail by the SV40 poly(A) signal. The reporter mRNA harbors the 5′UTR of the HIV-1 mRNA (pNL4.3 HIV clone, GenBank accession number AF 324493). The reporter also contains the defective encephalomyocarditis virus (ΔEMCV) 5′UTR that lacks IRES activity but avoid ribosomal readthrough from the first cistron (7,85). (**B**) HEK 293T cells were co-transfected with the dl HIV-1 IRES (300 ng) and different quantities (25–500 ng) of a plasmid encoding for a wt-hnRNP A1-HA protein. The presence of the endogenous hnRNP A1 and overexpressed wt-hnRNP A1-HA proteins was confirmed by western blot using GAPDH as loading control. (C, D). RLuc and FLuc activities were measured 24 h post-transfection, and data are presented as relative luciferase activity (RLA) (**C**), or as relative translational activity (RTA) (**D**). RTA corresponds to the FLuc/RLuc ratio that is used as an index of IRES activity. The RLA and RTA values obtained in the absence of wt-hnRNP A1-HA plasmid were set to 100%. Statistical analysis was performed by an ordinary two-way (**C**) or one-way (**D**) ANOVA test. (**E**, **F**) The dl HIV-1 IRES plasmid (200 ng) was co-transfected with the siA1-RNA (150 nM) or with the scRNA (150 nM). (**E**) Western blots were performed to detect the expression level of hnRNP A1 (48 h post-transfection) using GAPDH as a loading control. Dilutions (%) of the protein extract generated from the scRNA transfected cells were used as a relative measure to quantify the impact of the siA1 RNA. (**F**) RLuc and Fluc activities were measured 48 h post-transfection, and data are presented as RLA (%), with the values obtained in the presence of the scRNA set to 100%. Values represent the mean (±SEM) for three independent experiments, each conducted in duplicate. Statistical analysis was performed by an unpaired two-tailed *t*-test (*P<0.05). The relative level of total hnRNP A1 protein (%) in (**B**, **E**) was estimated based on the intensity of immunoreactive bands by imageJ.

Translation initiation of the HIV-1 full-length mRNA can follow a cap-dependent or an IRES-dependent mechanism (16,18,19,70). Next, we sought to determine if the effect of hnRNP A1 knockdown on HIV-1 full-length mRNA translation shown in Figure 1 was, at least in part, associated with HIV-1 IRES-mediated translation initiation as previously reported (10,23,36). For this, the dual-luciferase (dl), *Renilla* luciferase (RLuc) and firefly luciferase (FLuc) reporter plasmid dl HIV-1 IRES (Figure 2A) (7,19,29,33,34), was co-transfected in HEK 293T cells together with an irrelevant DNA (negative control) or different concentrations (25–500 ng) of a plasmid encoding for an HA-tagged hnRNP A1 (wild type (wt)-hnRNP A1-HA). The overexpression of the protein was confirmed by western blot (Figure 2B). The overexpression of hnRNP A1 did not affect cell viability (Supplemental Figure S1B). As the availability and compartmentalization of ITAFs affect IRES activity (71,72), we also analyzed the distribution of the wt-hnRNP A1-HA protein in cells. Western analysis of nuclear and cytoplasmic extracts confirmed that wt-hnRNP A1-HA was in both compartments, being, however, predominantly in the nucleus (Supplemental Figure S1C). Luciferase activities were measured, and data expressed as relative luciferase activity (RLA). As reported (10,23,36), the overexpression of hnRNP A1 increased FLuc activity without having a significant (*P*<0.05) impact over the expression RLuc (Figure 2C). Analysis of the FLuc/RLuc ratio (relative translational activity, RTA), index of IRES activity (7,9,12,13,29,32), better illustrates the significantly higher activity of the HIV-1 IRES when the wt-hnRNP A1-HA protein is overexpressed (50–500 ng DNA; Figure 2D). Also, a partial knockdown of the endogenous hnRNP A1 mRNA using the siA1-RNA, that reflected in a reduction ($\sim$30–40%) of protein levels (Figure 2E), was sufficient to significantly (*P* <0.05) reduce HIV-1 IRES activity (FLuc) without impacting on cap-dependent translation (RLuc) (Figure 2F). These observations, which are in agreement with a previous report (23), confirm that HIV-1 IRES-mediated translation initiation is highly dependent on hnRNP A1.

**Figure 3.**
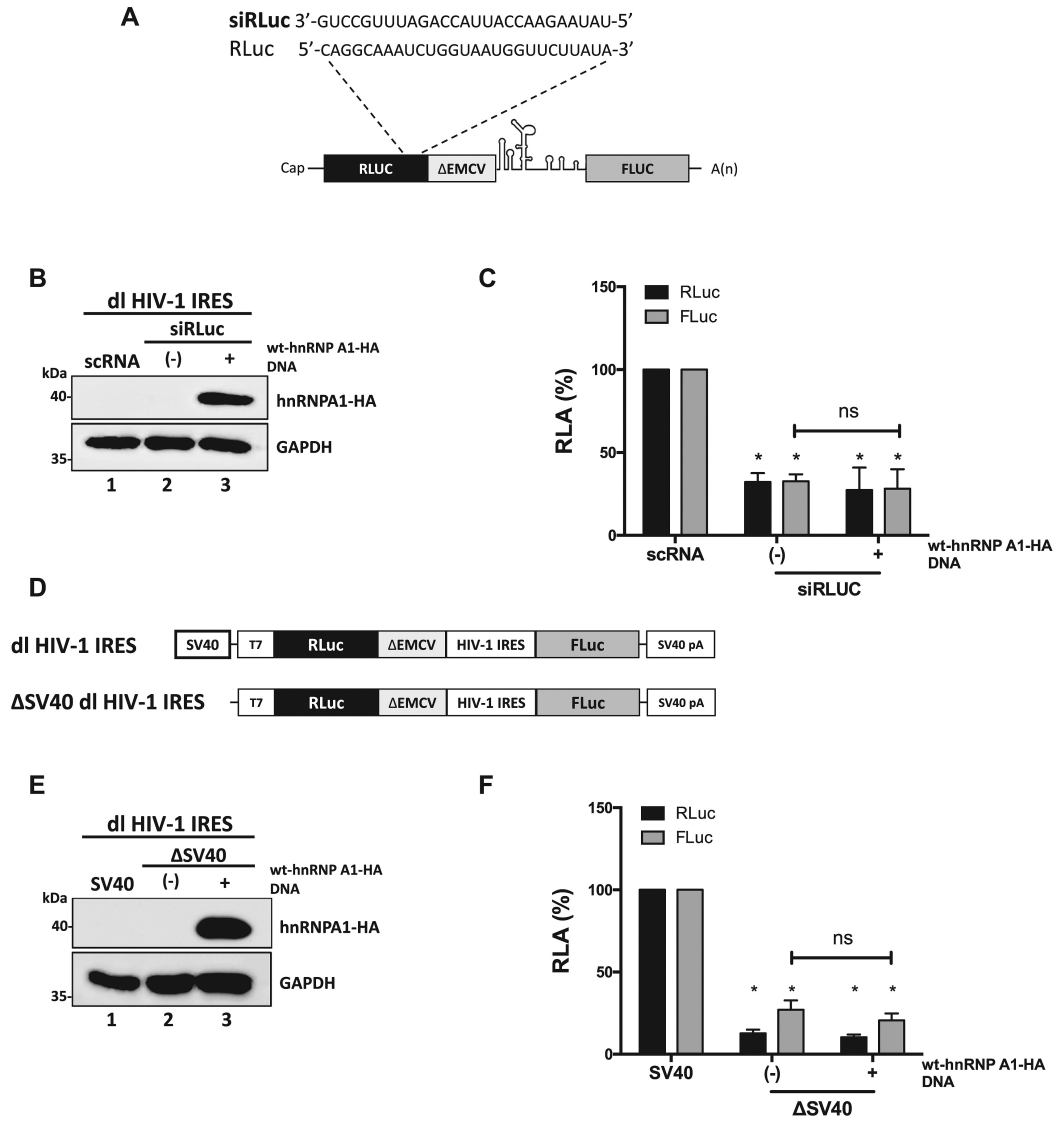
hnRNP A1 does not enhance alternative splicing of the dl HIV-1 IRES RNA nor increases the cryptic promoter activity of the dl HIV-1 IRES DNA. (**A**) Schematic representation of the dl reporter targeted by the siRLuc a siRNA targeting the *Renilla* luciferase ORF. (**B**, **C**) The dl HIV-1 IRES (150 ng) was co-transfected with a control scRNA (100 nM) or with siRLuc (100 nM), in the presence, or the absence, of the wt-hnRNP A1-HA (250 ng) plasmid. Total protein extracts were prepared 48 hrs post-transfection. (**B**) The expression of the wt-hnRNP A1-HA recombinant protein was determined by western blot, using the GAPDH protein as a loading control. (**C**) RLuc and FLuc activities were measured and expressed as RLA relative to the values obtained with scRNA, set to 100%. (**D–F**) HEK 293T cells were transfected with either the dl HIV-1 IRES (150 ng) or a promoterless ΔSV40-dl HIV-1 IRES (150 ng) vector (**D**) in the presence, or the absence (-), of the wt-hnRNP A1-HA (250 ng) plasmid. 24 hrs post-transfection total protein extracts were prepared. (**E**) The expression of the hnRNP A1-HA recombinant protein was determined by western blot, using the GAPDH protein as a loading control. (**F**) RLuc and FLuc activities were measured, and results are expressed as RLA relative to the activities obtained from the dl HIV-1 IRES vector when in the absence of the wt-hnRNP A1-HA, set to 100%. Values shown are the mean (±SEM) for three independent experiments, each performed in duplicate. Statistical analysis was performed by an ordinary two-way ANOVA test (*P <0.05; ns, not significant).

HnRNP A1 is involved in the regulation of HIV-1 RNA splicing (62,73–75). Furthermore, most of the wt-hnRNP A1-HA protein localized to the cell nucleus (Supplemental Figure S1C). Therefore, the possibility remained that the overexpression of hnRNP A1 induced the generation of a monocistronic mRNA encoding for a functional FLuc protein by alternative splicing. If so, the FLuc activity observed in Figure 2C–D would not reflect IRES-mediated translation initiation. To explore this possibility, the *Renilla* ORF of the dl HIV-1 IRES mRNA was targeted with an RLuc-siRNA (Figure 3A), as previously described (12,29,32). The RLuc-siRNA or a scRNA were co-transfected with the dl HIV-1 IRES in HEK 293T cells. The expression of the wt-hnRNP A1-HA protein was confirmed by western blot (Figure 3B, lane 3). In the presence of the RLuc-siRNA, both RLuc and FLuc activities were significantly (*P* <0.05) reduced, either when wt-hnRNP A1-HA was overexpressed ($\sim$72%) or not ($\sim$67%) (Figure 3C). When directly compared, the reduction of RLuc and FLuc induced by the RLuc-siRNA in the presence, or the absence, of wt-hnRNP A1-HA protein were not statistically different (Figure 3C). These observations indicated that RLuc and FLuc expression were associated with a single transcript.

The dl HIV-1 IRES reporter plasmid displays cell-type-specific cryptic promoter activity (10,29). To date, the cryptic promoter activity of the dl HIV-1 IRES vector has not been evaluated in HEK 293T cells. So next, we assessed if the overexpression of hnRNP A1 enhanced any alleged cryptic promoter activity present within the used dl-plasmid. For this, cells were transfected with the dl HIV-1 IRES plasmid together with a non-relevant DNA or the ΔSV40 dl HIV-1 IRES plasmid in the presence, or the absence, of the wt-hnRNP A1-HA plasmid. The ΔSV40 dl HIV-1 IRES plasmid lacks the SV40 promoter (Figure 3D) (29). The expression of the wt-hnRNP A1-HA protein was confirmed by western blot analysis (Figure 3E, lane 3). In the absence of the SV40 promoter (ΔSV40), both the RLuc and FLuc activities from the reporters were significantly (*P* <0.05) diminished in cells (Figure 3F). However, RLuc activity was detected, suggesting that in HEK 293T, a leakiness of ∼12% existed. Our results also showed that FLuc activity was 15% higher than RLuc (Figure 3F), suggesting the presence of a weak cryptic promoter (29). However, FLuc activity from the ΔSV40 dl HIV-1 IRES plasmid was reduced by more than 85%, suggesting that in HEK 293T cells, FLuc expression was mostly due to the presence of an IRES (Figure 3F). Furthermore, overexpression of wt-hnRNP A1-HA did not significantly increase FLuc expression from the ΔSV40 dl HIV-1 IRES plasmid, indicating that wt-hnRNP A1-HA does not enhance cryptic promoter activity from the used vector in HEK 293T cells (Figure 3F). Together, these results confirmed that hnRNP A1 acts as an ITAF for the HIV-1 IRES in HEK 293T cells.

### ITAF activity of hnRNP A1 on the HIV-1 IRES is modulated by post-translational modifications

**Figure 4.**
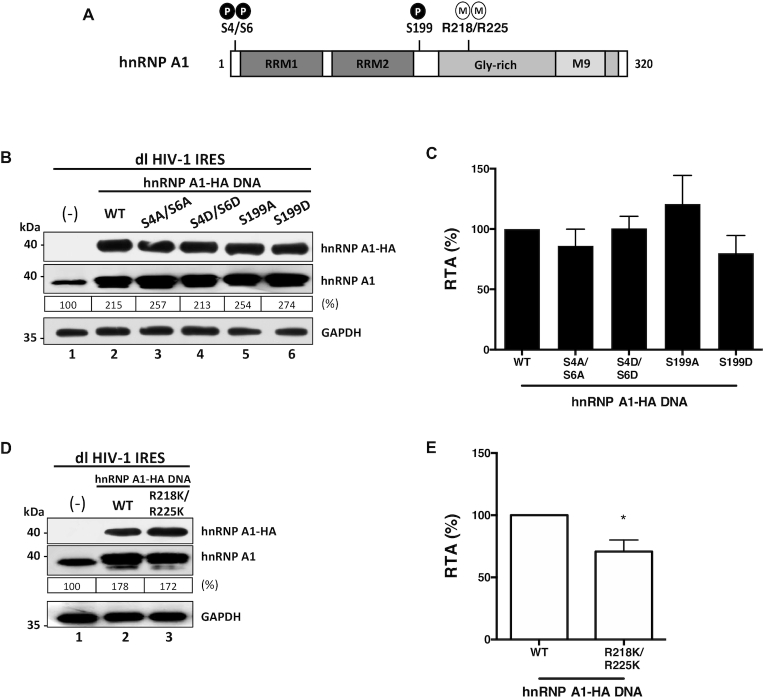
hnRNP A1 post-translational modifications impact on HIV-1 IRES activity. (**A**) hnRNP A1-HA mutants for the phosphorylation sites S4/S6 and S199, or methylation sites R218/R225 were generated by site-directed mutagenesis of the wt-hnRNP A1-HA template plasmid. (**B**–**E**) HEK 293T cells were co-transfected with the dl HIV-1 IRES (300 ng) together with each hnRNP A1-HA (500 ng) plasmid. Total protein extracts were prepared 24 h post-transfection. (**B**, **D**) Western blots were performed to detect the expression level of total hnRNP A1 and hnRNP A1-HA proteins using GAPDH as a loading control. (**C**, **E**) RLuc and FLuc activities were measured, and results are presented as RTA, relative to the activities in the presence of the wt-hnRNP A1-HA, set to 100%. Values shown are the mean (±SEM) for three independent experiments, each performed in duplicate. Statistical analysis was performed by an unpaired two-tailed *t*-test (*P<0.05). The relative level of total hnRNP A1 protein, PRMT5 and SDMA (%) in (**B**, **D**) was estimated based on the intensity of immunoreactive bands by image-J.

Treatment of cells with inhibitors of the serine/threonine kinases Mnk selectively reduced HIV-1 IRES activity without impacting on cap-dependent translation initiation (23). This observation suggested that Mnk-associated phosphorylations of hnRNP A1, at serines (S) 308-313/316 (76), are required to stimulate the activity from the HIV-1 IRES (23). We wondered if other hnRNP A1 phosphorylation sites, known to alter the protein's ability to act as an ITAF for cellular IRESs (42,50), were also implicated in the modulation of the HIV-1 IRES (Figure 4A). For this, HEK 293T cells were transfected with the dl HIV-1 IRES vector together with an empty control DNA or plasmids encoding for wt-hnRNP A1-HA, the alanine (A)-substitution mutants hnRNP A1-HA S4A/S6A, hnRNP A1-HA S199A, the corresponding aspartic acid (D)-phosphomimetic mutants hnRNP A1-HA S4D/S6D, or hnRNP A1-HA S199D. Expression of the total hnRNP A1 and the HA-tagged proteins was monitored by western blot (Figure 4B). Luciferase activities were determined and results presented as RTA (Figure 4C), showed that the mutants hnRNP A1-HA S4A/S6A ($\sim$86%), hnRNP A1-HA S4D/S6D ($\sim$101%) and the hnRNP A1-HA S199D ($\sim$80%) stimulated HIV-1 IRES activity as did the wt-hnRNP A1-HA protein (100% increase) (Figure 4C). So, neither the S6K2- nor Akt-dependent phosphorylation of hnRNP A1 influenced the HIV-1 IRES activity.

Next, we questioned whether other PTMs such as PRMT5-induced symmetrical dimethylation of arginine (SDMA) residues in hnRNP A1, R218 and R225, known to regulate cellular IRESs (41), controlled HIV-1 IRES activity (Figure 4A). For this, the dl HIV-1 IRES plasmid was co-transfected in HEK 293T cells with an irrelevant control DNA, or plasmids encoding for the wt-hnRNP A1-HA or the hnRNP A1-HA R218/225K double lysine-substitution mutant, reported being refractory to PRMT5 methylation (41). The reduced methylation of the hnRNP A1-HA R218/225K double lysine-substitution mutant in cells was confirmed (Supplemental Figure S2). The expression of the total hnRNP A1 and the HA-tagged proteins was monitored by western blotting (Figure 4D). The results presented as RTA, showed that the hnRNP A1-HA R218/225K mutant exhibited a reduced ability to stimulate HIV-1 IRES activity ($\sim$70%) when compared to the wt-hnRNP A1-HA ($\sim$100% increase) (Figure 4E). Thus, as reported for cellular IRESs (41), the PRMT5-induced methylations of hnRNP A1 at positions R218 and R225 would enable the ITAF to up-regulate the HIV-1 IRES.

### Pharmacological inhibition of PRMT5 negatively impacts on HIV-1 IRES activity

The above observations suggested that PRMT5-induced symmetric arginine demethylation of hnRNP A1 regulates HIV-1 IRES-mediated translation initiation. If so, the pharmacological inhibition of PRMT5 should negatively impact on the HIV-1 IRES activity. To evaluate this, we used EPZ015666, a specific substrate-competitive PRMT5 inhibitor (57). In the time frame of the experiment, the treatment of cells with EPZ015666 (5–20 μM) did not impact on their viability (Supplemental Figure S3A), nor did it reduce global cellular translation, evaluated by Surface Sensing of Translation (SUnSET) (56) (Supplemental Figure S3B). As an additional control, cell extracts obtained from HEK 293T expressing RLuc and FLuc from the dl HIV-1 IRES plasmid were mixed, or not, with EPZ015666 (20 μM) and luciferase activities were determined. The PRMT5 inhibitor did not affect RLuc or FLuc enzymatic activity (Supplemental Figure S3C).

**Figure 5.**
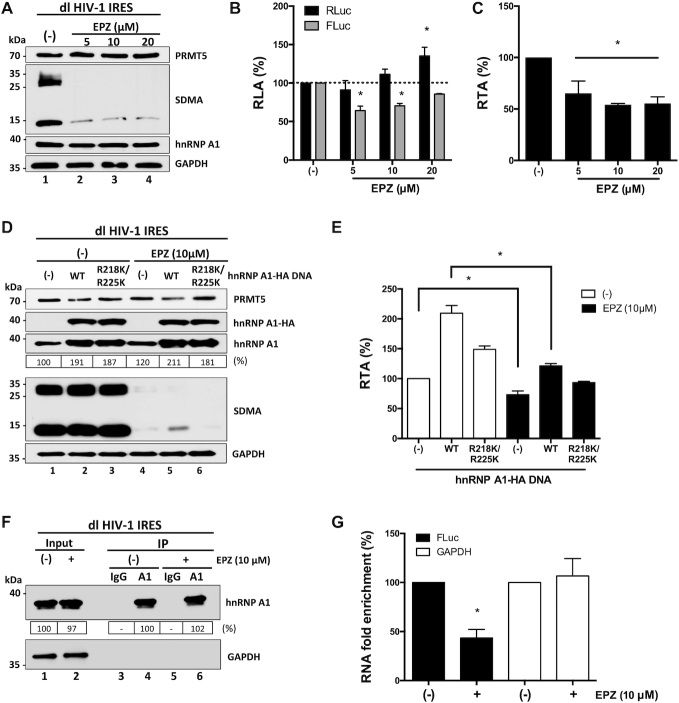
Pharmacological inhibition of PRMT5 methyltransferase activity impairs HIV-1 IRES activity. (**A**–**C**) HEK 293T **c**ells were transfected with the dl HIV-1 IRES (300 ng) plasmid and treated, or not (only vehicle; (–)), with EPZ015666 (5, 10 or 20 μM), a specific inhibitor of the PRMT5 activity (57). Total protein extracts were prepared 48 h post-transfection and treatment. (**A**) The PRMT5 activity (SDMA), and levels of PRMT5 and hnRNP A1 proteins in extracts from untreated cell (lane 1) or from cells teated with at the different drug concentrations (5, 10, or 20 μM) were evaluated by western blot using GAPDH as a loading control. (**B**, **C**) RLuc and FLuc activities were measured, and data are presented as RLA (**B**) or RTA (**C**) relative to the non-treated (–) cells, set to 100%. Statistical analysis was performed by an ordinary two-way (**B**) or one-way (**C**) ANOVA test (*P <0.05). (**D**, **E**) The dl HIV-1 IRES (300 ng) plasmid was co-transfected, or not ((-); lanes 1 and 4), with the plasmid encoding the wt-hnRNP A1-HA (500 ng; lanes 2 and 5) or hnRNP A1-HA R218K/R225K mutant (500 ng; lanes 3 and 6) protein in the absence (only vehicle (–); lanes 1, 2 and 3) or the presence of EPZ015666 (10 μM; lanes 4, 5 and 6). Total protein extracts were prepared 48 h post-transfection and treatment. (**D**) The levels of PRMT5, hnRNP A1 and hnRNP A1-HA proteins, and PRMT5 activity (SDMA) for each combination was followed by western blot using GAPDH as a loading control. (**E**) RLuc and FLuc activities were determined, and data are presented as RTA relative to the wt-hnRNP A1-HA untransfected (–), and untreated condition set to 100%. Statistical analysis was performed by an ordinary one-way ANOVA test (*P < 0.05). Values shown are the mean (+/- SEM) for three independent experiments, each performed in duplicate. (**F**, **G**) HEK 293T cells were transfected with the dl HIV-1 IRES plasmid and cells were treated (+), or not (–), with EPZ015666 (10 μM). Proteins and RNAs were ultraviolet (UV) cross-linked (254 nm-irradiated with 400 mJ/cm^2^). Endogenous hnRNP A1 was immunoprecipitated (IP) from cell extracts using protein A/G-agarose (PGA)-beads loaded with an anti-hnRNP A1 antibody or using PGA-beads loaded with an anti-IgG antibody as a negative control. (**F**) The hnRNP A1 protein present in the input extracts and the IPs were evaluated by western blotting using an anti-hnRNP A1 antibody. The protein A/G HRP conjugated was used as the secondary antibody. (**G**) The quantity of FLuc encoding RNA (FLuc) or GAPDH encoding RNA (GAPDH) covalently bound to hnRNP A1 in EPZ015666 treated (+) or untreated (–) cells after the IP was determined by an RT-qPCR assay as described in (35). RNA fold enrichment, as defined in (35), obtained in the IP from untreated cells, was set to 100%. Values shown are the mean (±SEM) for three independent experiments; each RT-qPCR assay was performed in duplicate. Statistical analyses were performed using the ANOVA Kruskal–Wallis test (*P* <0.05). The relative level of total hnRNP A1 protein (%) in (**D**, **F**) was estimated based on the intensity of immunoreactive bands by image-J.

Next, HEK 293T cells were transfected with the dl HIV-1 IRES plasmid and treated, or not (only vehicle), with different concentrations of EPZ015666 (5–20 μM). As evidenced by western blotting (41), the treatment of cells with EPZ015666 (5–20 μM) reduced SDMA without an evident impact on endogenous PRMT5 or hnRNP A1 protein levels (Figure 5A). Luciferase activities were determined using the same total protein extract and expressed as RLA. RLuc activity in EPZ015666 treated cells showed an increasing trend reaching a significant (*P* <0.05) enhancement of activity ($\sim$35% increase) at the highest concentration of the drug (20 μM) (Figure 5B). In contrast, the FLuc activity was significantly decreased ($\sim$35% reduction) at the lowest concentrations of EPZ015666 (5 μM) (Figure 5B). Results are also presented as RTA to illustrate better the significantly (*P* <0.05) lower activity ($\sim$35–45% reduction) of the HIV-1 IRES in cells treated with EPZ015666 (Figure 5C). So, while RLuc activity is maintained, or increased, FLuc activity is reduced in the presence of the PRMT5 inhibitor, suggesting that HIV-1 IRES-mediated translation but not cap-mediated initiation is dependent on PRMT5 activity.

To further assess a possible link between EPZ015666, PRMT5-methylations, hnRNP A1, and the activity of the HIV-1 IRES, HEK 293T cells were transfected with the dl HIV-1 IRES plasmid together with an irrelevant DNA, or plasmids encoding for the wt-hnRNP A1-HA or hnRNP A1-HA R218/225K. Transfected cells were then treated, or not, with EPZ015666 (10 μM) (Figure 5A-C and (41,57,58)). Expression of wt-hnRNP A1-HA, hnRNP A1-HA R218/225K, total hnRNP A1, and PRMT5 proteins, as well as SDMA, were followed by western blotting (Figure 5D). As expected, the treatment of cells with EPZ015666 reduced SDMA (Figure 5D) without an evident impact on the levels of expression of the endogenous PRMT5 or hnRNP A1 proteins (Figure 5D). RLuc and FLuc activities were determined using the same protein extract, and data expressed as RTA (Figure 5E). In drug-untreated cells (Figure 5E, white bars), overexpression of wt-hnRNP A1-HA increased HIV-1 IRES activity (by $\sim$110%), as did, yet to a much lower extent, the overexpression of the hnRNP A1-HA R218/225K mutant ($\sim$48%). These findings confirmed our previous observations (Figure 4B), by showing that hnRNP A1-HA R218/225K mutant stimulates the HIV-1 IRES to a lesser extent than the wt-hnRNP A1-HA (Figure 5E). Treatment of transfected cells with EPZ015666 (10 μM; Figure 5E, black bars) significantly (*P* <0.05) reduced (by $\sim$30%) HIV-1 IRES activity in cells transfected with the control DNA, confirming that the activity of endogenous PRMT5 is required for optimal HIV-1 IRES activity (compare (–) white and (–) black bars in Figure 5E). In cells expressing the wt-hnRNP A1-HA and treated with the drug, a marginal $\sim$20% increase in HIV-1 IRES activity was observed, suggesting that EPZ015666 significantly (*P* <0.05) hindered (by $\sim$80–90%) the ability of wt-hnRNP A1-HA to stimulate HIV-1 IRES activity. In the presence of the drug, the overexpression of the hnRNP A1-HA R218/225K mutant reduced HIV-1 IRES activity ($\sim$7%), suggesting that the endogenous hnRNP A1 contributes to the increase in HIV-1 IRES activity observed when the hnRNP A1-HA R218/225K mutant is overexpressed in the absence of the drug (Figure 5E). So, the treatment of cells with EPZ015666 abolished the ability of wt-hnRNP A1-HA to stimulate HIV-1 IRES activity when overexpressed (compare wt, white and black bars in Figure 5E). Thus, the enhancement of HIV-1 IRES activity by hnRNP A1 requires the protein to be methylated by PRMT5.

The arginine sites R218 and R225 of hnRNP A1 are located in the glycine–arginine-rich (Gly-rich) motif, responsible for protein-protein interactions and RNA binding (41,77). Reports show that PRMT5-induced SDMA enhances the ability of hnRNP A1 to engage its target mRNAs (41,78). So, next, we used an ultraviolet (UV) cross-linking immunoprecipitation (CLIP) assay coupled with a reverse transcription (RT)-quantitative polymerase chain reaction (qPCR) analysis to determine if PRMT5 induced SDMA of hnRNP A1 altered the binding of the ITAF to the dl HIV-1 IRES mRNA. For this, HEK 293T cells were transfected with the dl HIV-1 IRES plasmid and cells were treated, or not, with EPZ015666 (10 μM). Proteins and RNAs were covalently link using UV light, and the endogenous hnRNP A1 was immunoprecipitated (IP) from extracts using protein A/G-agarose (PGA)-beads loaded with an anti-hnRNP A1 antibody (A1 in Figure 5F). PGA-beads loaded with an anti-IgG antibody was used as a negative control (IgG in Figure 5F). The presence of hnRNP A1 in input extracts and IPs was confirmed by western blotting (Figure 5F, lanes 1 and 2). A comparison of GAPDH in the input suggests that similar amounts of protein were loaded (Figure 5F, lanes 1 and 2). Treatment of cells with EPZ015666 did not reduce the levels of hnRNP A1 recovered by IP (Figure 5F, lanes 4 and 6). The quantity of FLuc encoding RNA (FLuc RNA) or GAPDH encoding RNA (GAPDH RNA) covalently bound to hnRNP A1 in EPZ015666 treated or untreated cells after the IP was quantified by an RT-qPCR assay (Figure 5G) (35). Treatment of cells with EPZ015666 significantly reduced (57%) the amount of FLuc encoding RNA associated with hnRNP A1 (Figure 5G; black bar). Since the dl HIV-1 IRES RNA is the only source of FLuc RNA in cells, we conclude that PRMT5 methylation regulates hnRNP A1-RNA binding to its target RNA fact that directly correlates with the activity of the HIV-1 IRES.

### PTMs of hnRNP A1 modulate the HTLV-1 IRES

**Figure 6.**
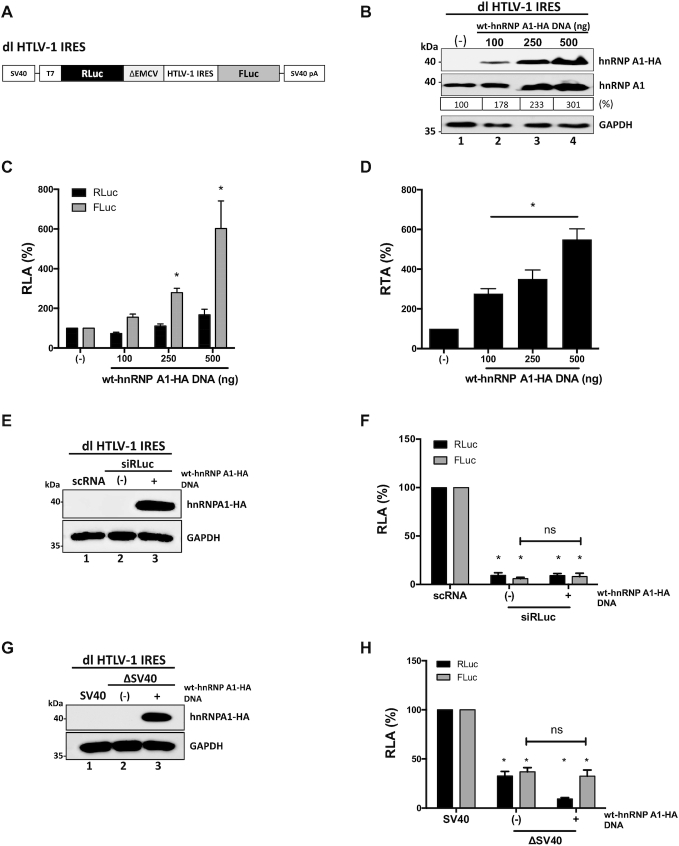
HnRNP A1 is an ITAF for the HTLV-1 IRES. (**A**) Schematic representation of the dl HTLV-1 IRES plasmid described in (12). (**B**–**D**) HEK 293T cells were co-transfected with the dl HTLV-1 IRES (300 ng) and increasing amounts of the wt-hnRNP A1-HA (100, 250, 500 ng) plasmid. Total protein extracts were prepared 24 h post-transfection. (**B**) Western blots were performed to follow the expression level of total hnRNP A1 and wt-hnRNP A-HA proteins using GAPDH as a loading control. The relative level of hnRNP A1 protein (%) was estimated based on the intensity of immunoreactive bands by imageJ. (**C**, **D**) RLuc and FLuc activities were measured, and data are presented as RLA (**C**) or RTA (**D**). The RLA and RTA values obtained when in the absence (–) of transfected wt-hnRNP A1-HA were set to 100%. Statistical analysis was performed by an ordinary two-way (**C**) or one-way (**D**) ANOVA test (*P <0.05). (**E**, **F**) The dl HTLV-1 IRES (150 ng) was co-transfected with a control scRNA (100 nM) or with siRLuc (100 nM), in the presence (+), or the absence (–), of the wt-hnRNP A1-HA (250 ng) plasmid. Total protein extracts were prepared 48 h post-transfection. (**E**) The expression of the hnRNP A1-HA recombinant protein was determined by western blot, using the GAPDH protein as a loading control. (**F**) RLuc and FLuc activities were measured and expressed relative to the values obtained with scRNA, set to 100% (RLA). (**G**, **H**) HEK 293T cells were transfected with either the dl HTLV-1 IRES (150 ng) or a promoterless ΔSV40-dl HTLV-1 IRES (150 ng) vector in the presence (+), or the absence (–), of the wt-hnRNP A1-HA (250 ng) plasmid. Total protein extracts were prepared 24 h post-transfection. (**G**) The expression of the hnRNP A1-HA recombinant protein was determined by western blot, using the GAPDH protein as a loading control. (**H**) RLuc and FLuc activities were measured, and results are expressed as RLA relative to the activities obtained from the dl HTLV-1 IRES plasmid transfected in the absence of the wt-hnRNP A1-HA, set to 100%. Values shown are the mean (±SEM) for three independent experiments, each performed in duplicate. Statistical analysis was performed by an ordinary two-way ANOVA test (*P <0.05; ns, not significant).

Next, we wondered whether the hnRNP A1 PTMs also modulated the activity of other retroviral IRESs. To address this question, we first focused on the IRES present within the 5′UTR of the full-length mRNA of HTLV-1 (11,12). The dl HTLV-1 IRES plasmid that harbors the HTLV-1 IRES in the same bicistronic backbone as the dl HIV-1 IRES (Figure 6A) (4,9), was co-transfected in HEK 293T cells with a control DNA or with different concentrations (100–500 ng) of the wt-hnRNP A1-HA plasmid. The expression of the HA-tagged and total hnRNP A1 proteins was confirmed by western blot (Figure 6B). Luciferase activities were measured, and data were expressed as RLA (Figure 6C). Overexpression of wt-hnRNP A1-HA did not significantly alter cap-dependent translation (RLuc), while the activity from the HTLV-1 IRES was significantly (*P*<0.05) increased (up to a $\sim$500% increase) (Figure 6C). The impact of hnRNP A1 overexpression over the HTLV-1 IRES is better appreciated when data are presented as RTA (Figure 6D). These observations indicated that hnRNP A1 acts as an ITAF for the HTLV-1 IRES.

Next, the dl HTLV-1 IRES mRNA was targeted with the siRLuc RNA in the presence or the absence of the recombinant wt-hnRNAP A1-HA protein (12,29,32). The expression of the recombinant protein was confirmed by western blot (Figure 6E, lane 3). In the presence of the siRLuc RNA, both RLuc and FLuc activities were significantly (*P* <0.05) reduced, either when wt-hnRNP A1-HA was overexpressed, or not (Figure 6E and F). When directly compared, the reduction of RLuc and FLuc induced by the RLuc-siRNA in the presence or the absence of wt-hnRNP A1-HA protein were not statistically different (Figure 6F). These results indicated that RLuc and FLuc expression was associated with a single transcript.

Then, HEK 293T cells were transfected with the dl HTLV-1 IRES plasmid or with the ΔSV40 dl HTLV-1 IRES plasmid (12), in the presence or the absence of the wt-hnRNP A1-HA plasmid. In agreement with previous reports (12,29), and results presented in Figure 3F, a cryptic promoter activity from the dl HTLV-1 IRES plasmid was observed (Figure 6H). Nonetheless, the overexpression of wt-hnRNP A1-HA confirmed by western blot (Figure 6G, lane 3), did not further enhance cryptic promoter activity from the dl HTLV-1 IRES vector in HEK 293T cells (Figure 6H). This observation suggests that the increase in FLuc activity observed in Figure 6C is most probably due to an enhancement of HTLV-1 IRES activity induced by hnRNP A1 overexpression.

**Figure 7.**
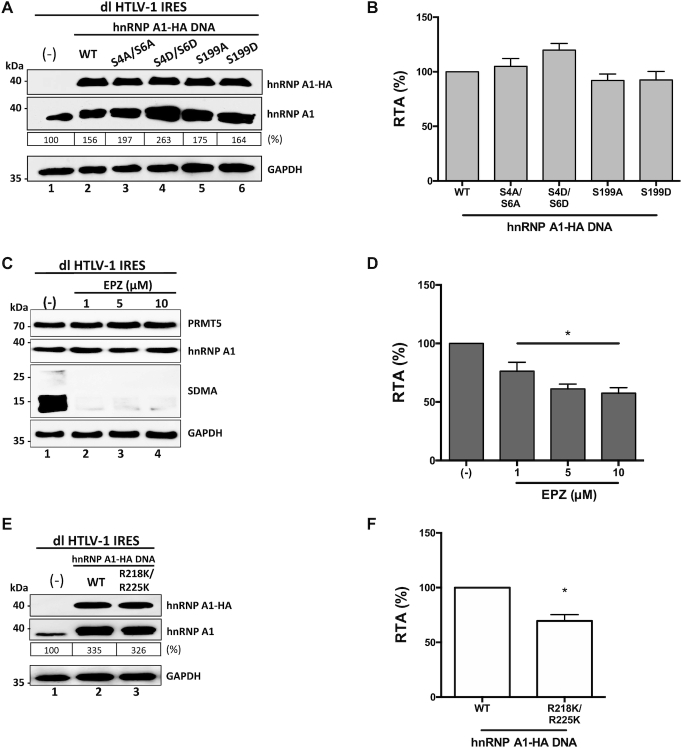
HTLV-1 IRES activity is stimulated by PRMT5 activity and PRMT5-associated hnRNP A1 methylations. (**A-B**) hnRNP A1-HA mutant plasmids (500 ng) for the phosphorylation sites S4/S6 and S199 (indicated in the x-axis) were co-transfected with the dl HTLV-1 IRES plasmid (300 ng). Total protein extracts were prepared 24 hrs post-transfection. (**A**) Western blots were performed to detect the expression level of total hnRNP A1 and hnRNP A1-HA proteins using GAPDH as a loading control. (**B**) RLuc and FLuc activities were measured, and results are presented as RTA, relative to the activities obtained from the dl HTLV-1 IRES vector in the presence of the wt-hnRNP A1 protein, set to 100%. Statistical analysis was performed by an ordinary one-way ANOVA test (*P <0.05). (**C**, **D**) Cells transfected with the dl HTLV-1 IRES (300 ng) plasmid were treated, or not (–), with EPZ015666 (1, 5 or 10 μM). Total protein extracts were prepared 48 hrs post-transfection and treatment. (**C**) The levels of PRMT5 and hnRNP A1 proteins and PRMT5 activity (SDMA) were determined by western blot using GAPDH as a loading control. (**D**) RLuc and FLuc activities were measured, and data are presented as RTA relative to the untreated (−) condition set to 100%. Statistical analysis was performed by an ordinary one-way ANOVA test (*P<0.05). (**E**, **F**) The dl HTLV-1 IRES (300 ng) was co-transfected with the wt-hnRNP A1-HA (500 ng) or hnRNP A1-HA R218K/R225K (500 ng) encoding plasmids. Total protein extracts were prepared 24 h post-transfection. (**E**) Western blots were performed to detect the levels of endogenous and overexpressed hnRNP A1-HA proteins, using GAPDH as a loading control. (**F**) RLuc and FLuc activities were measured, and data are presented as RTA relative to the wt-hnRNP A1-HA, set to 100%. Statistical analysis was performed by an unpaired two-tailed *t*-test (*P<0.05). Values shown are the mean (±SEM) for three independent experiments, each performed in duplicate. The relative level of total hnRNP A1 protein (%) in (**A**, **E**) was estimated based on the intensity of immunoreactive bands by imageJ.

Having determined that hnRNP A1 is an ITAF for the HTLV-1 IRES, we then sought to establish if PTMs of the protein altered the activity of the HTLV-1 IRES. For this, HEK 293-T cells were transfected with the dl HTLV-1 IRES vector together with plasmids encoding for wt- hnRNP A1-HA, or mutants hnRNP A1-HA S4A/S6A, hnRNP A1-HA S4D/S6D, hnRNP A1-HA S199A or hnRNP A1-HA S199D. Western analysis confirmed the expression of total and HA-tagged hnRNP A1 proteins (Figure 7A). Luciferase activities were determined using the same protein extracts, and the RTA was calculated. Results showed that all mutants enhance the HTLV-1 IRES to levels similar to that of the wt-hnRNP A1-HA protein (Figure 7B). So, neither the S6K2- nor Akt-dependent phosphorylation of hnRNP A1 influenced the HTLV-1 IRES activity.

Next, we wondered whether PRMT5-induced SDMA regulated HTLV-1 IRES activity. So, HEK 293T cells were transfected with the dl HTLV-1 IRES plasmid followed by the treatment, or not, with EPZ015666. SDMA and protein expression was monitored by western blotting (Figure 7C). The drug reduced SDMA without an evident impact on PRMT5 or hnRNP A1 protein levels (Figure 7C). RLuc and FLuc activities were determined using the same total protein extracts, and results were expressed as RTA (Figure 7D). HTLV-1 IRES-mediated translation initiation was significantly (*P* <0.05) reduced ($\sim$24–43%) in the presence of the EPZ015666 (Figure 7B), suggesting that PRMT5 induced SDMA are required for optimal HTLV-1 IRES activity. To determine the role of PRMT5-induced SDMA of hnRNP A1 on HTLV-1 IRES activity, the dl HTLV-1 IRES plasmid was co-transfected in HEK 293T cells with an irrelevant control DNA, or plasmids encoding for wt-hnRNP A1-HA or the hnRNP A1-HA R218/225K recombinant proteins. Expression of the total hnRNP A1 and the HA-tagged proteins was confirmed by western blotting (Figure 7E). RLuc and FLuc activities were determined, and data presented as RTA, showed that the hnRNP A1-HA R218/225K mutant increase HTLV-1 IRES activity significantly less ($\sim$30% reduction) than the wt-hnRNP A1-HA (Figure 7F). Therefore, PRMT5-induced methylation of hnRNP A1 at positions R218/225 enables the ITAF to up-regulated HTLV-1 IRES activity. Together results show that hnRNP A1 PTMs alter the HTLV-1 IRES activity in a similar way than was observed for the HIV-1 IRES.

### PTMs of hnRNP A1 modulate the MMTV IRES

**Figure 8.**
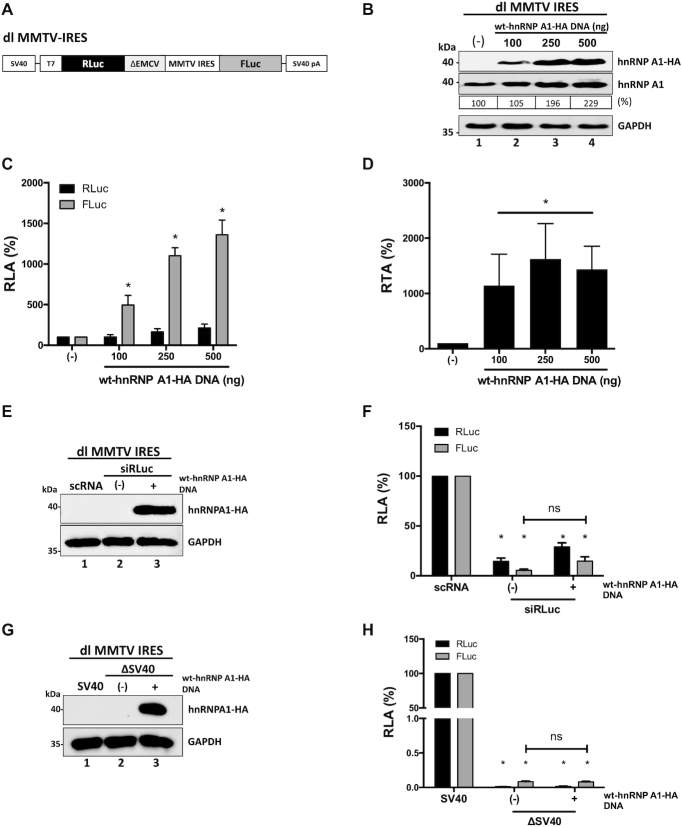
HnRNP A1 is an ITAF for the MMTV IRES. (**A**) Schematic representation of the dl MMTV IRES plasmid described in (13). (B−D) HEK 293T cells were co-transfected with the dl MMTV IRES plasmid (300 ng) and increasing amounts (100, 250, 500 ng) of the wt-hnRNP A1-HA plasmid. Total protein extracts were prepared 24 hrs post-transfection. (**B**) Western blots were performed to detect the expression level of total hnRNP A1 and hnRNP A1-HA proteins using GAPDH as a loading control. The relative level of hnRNP A1 protein (%) was estimated based on the intensity of immunoreactive bands by imageJ. (**C**, **D**) RLuc and FLuc activities were measured, and data are presented as RLA (**C**) or RTA (**D**). The RLA and RTA values in the absence of transfected wt-hnRNP A1-HA were set to 100%. Statistical analysis was performed by an ordinary two-way (**C**) or one-way (**D**) ANOVA test (*P < 0.05). (**E**, **F**) The dl MMTV IRES (150 ng) was co-transfected with a control scRNA (100 nM) or with siRLuc (100 nM), in the presence (+), or the absence (−), of the wt-hnRNP A1-HA (250 ng) plasmid. Total protein extracts were prepared 48 hrs post-transfection. (**E**) The expression of the hnRNP A1-HA recombinant protein was determined by western blot, using the GAPDH protein as a loading control. (**F**) RLuc and FLuc activities were measured and expressed relative to the values obtained with scRNA, set to 100% (RLA). (**G**, **H**) HEK 293T cells were transfected with either the dl MMTV IRES (150 ng) or a promoterless ΔSV40-dl MMTV IRES (150 ng) vector in the presence (+), or the absence (−), of the wt-hnRNP A1-HA (250 ng) plasmid. Total protein extracts were prepared 24 h post-transfection. (**G**) The expression of the hnRNP A1-HA recombinant protein was determined by western blot, using the GAPDH protein as a loading control. (**H**) RLuc and FLuc activities were measured, and results are expressed as RLA relative to the activities obtained from the dl MMTV IRES plasmid transfected in the absence of the wt-hnRNP A1-HA, set to 100%. Values shown are the mean (±SEM) for three independent experiments, each performed in duplicate. Statistical analysis was performed by an ordinary two-way ANOVA test (*P < 0.05; ns, not significant).

We also evaluated whether hnRNP A1 regulated the IRES present within the 5′UTR of the mouse mammary tumor virus (MMTV) genomic RNA (13). To this end, the dl MMTV IRES plasmid (Figure 8A), which harbors the MMTV IRES in the same bicistronic backbone as the dl HIV-1 IRES (7,13), and dl HTLV-1 IRES (12), was co-transfected in HEK 293T cells with a control DNA or with different concentrations (100–500 ng) of the wt-hnRNP A1-HA plasmid. The expression of the hnRNP A1 proteins was confirmed by western blot (Figure 8B). Luciferase activities were measured and expressed as RLA (Figure 8C) or RTA (Figure 8D). Results show that the overexpression of hnRNP A1 significantly (*P* <0.05) enhanced ($\sim$400–1360% increase) MMTV IRES activity without significantly affecting RLuc activity (Figure 8C). The enhancement of MMTV IRES activity is better evidenced when data are shown as RTA (Figure 8D). Results suggested that hnRNP A1 is also an ITAF for the MMTV IRES. Next, the dl MMTV IRES mRNA was targeted with the siRLuc RNA in the presence or the absence of the recombinant wt-hnRNAP A1-HA protein (12,29,32). The expression of the recombinant protein was confirmed by western blot (Figure 8E, lane 3). In the presence of the RLuc-siRNA, both RLuc and FLuc activities were significantly (*P* <0.05) reduced, either when wt-hnRNP A1-HA was overexpressed or not (Figure 8F). When directly compared, the reduction of RLuc and FLuc induced by the RLuc-siRNA in the presence or the absence of wt-hnRNP A1-HA protein were not statistically different (Figure 8F). The enhancement of cryptic promoter activity by the overexpression of hnRNP A1 was also evaluated. For this, HEK 293T cells were transfected with the dl MMTV IRES plasmid or with the ΔSV40 dl MMTV IRES plasmid (13), in the presence or the absence of the wt-hnRNP A1-HA plasmid. Results show that the overexpression of wt-hnRNP A1-HA confirmed by western blot (Figure 8G. lane 3), did not enhance cryptic promoter activity from the dl MMTV IRES vector in HEK 293T cells (Figure 8H). We, therefore, conclude that hnRNP A1 is an ITAF for the MMTV IRES.

**Figure 9.**
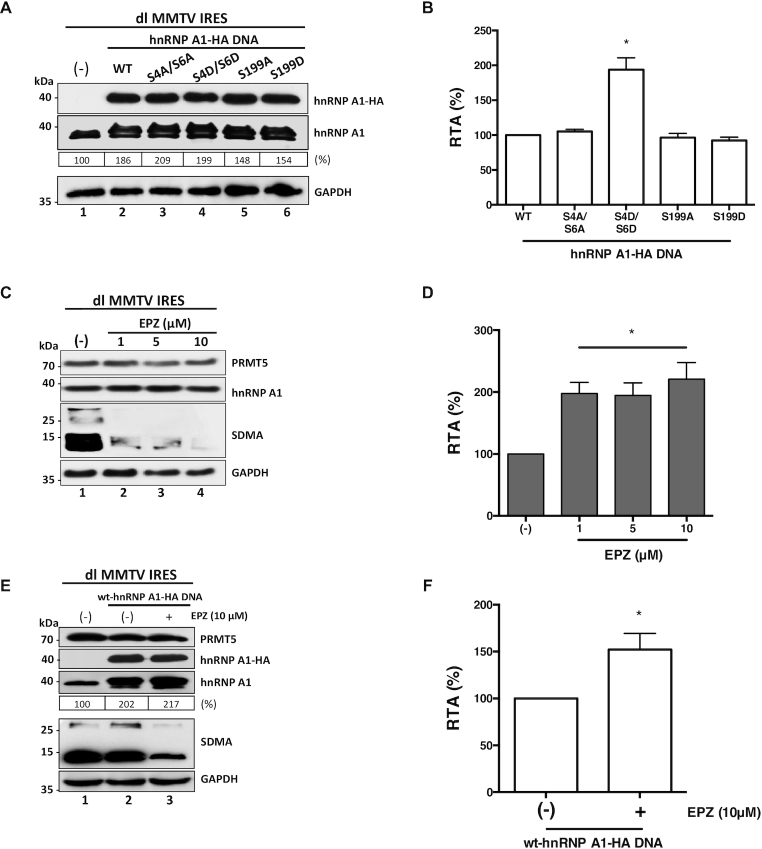
MMTV IRES activity is stimulated by S6K2-associated hnRNP A1 phosphorylations but inhibited by PRMT5 activity and PRMT5-induced hnRNP A1 methylations. (**A**, **B**) hnRNP A1-HA S4/S6 or S199 mutant plasmids (500 ng) were co-transfected with the dl MMTV IRES plasmid (300 ng). Total protein extracts were prepared 24 h post-transfection. (**A**) Western blots were performed to detect the expression level of total hnRNP A1 and hnRNP A1-HA proteins using GAPDH as a loading control. (**B**) RLuc and FLuc activities were measured, and results are presented as RTA, relative to the activities obtained from the dl MMTV IRES vector in the presence of the wt-hnRNP A1-HA protein, set to 100%. Statistical analysis was performed by an ordinary one-way ANOVA test (*P < 0.05). (**C**, **D**) Cells transfected with the dl MMTV IRES (300 ng) plasmid were treated, or not (-), with of EPZ015666 (1, 5 or 10 μM). Total protein extracts were prepared 48 hrs post-transfection and treatment. (**C**) The levels of PRMT5 and hnRNP A1 protein, and PRMT5 activity (SDMA) were determined by western blot using GAPDH as a loading control. (**D**) RLuc and FLuc activities were measured, and data are presented as RTA relative to the untreated (−) condition set to 100%. Statistical analysis was performed by an ordinary one-way ANOVA test (*P < 0.05). (**E**, **F**) Cells were co-transfected with the dl MMTV IRES (300 ng) and the wt-hnRNP A1-HA (500 ng; lanes 2 and 3) plasmids and treated (+), or not (−), with 10 μM of EPZ015666. Total protein extracts were prepared 48 hrs post-transfection and treatment. (**E**) Western blots were performed to detect the levels of PRMT5 and PRMT5 activity (SDMA), hnRNP A1, hnRNP A1-HA proteins, and GAPDH as a loading control. (**F**) RLuc and FLuc activities were measured, and data are presented as RTA relative to the drug untreated (−) condition set to 100%. Statistical analysis was performed by an unpaired two-tailed *t*-test (*P < 0.05). Values shown are the mean (±SEM) for three independent experiments, each performed in duplicate. The relative level of hnRNP A1 protein (%) in (**A**, **E**) was estimated based on the intensity of immunoreactive bands by imageJ.

Next, we evaluated the effect of hnRNP A1 PTMs on MMTV IRES activity. For this, HEK 293T cells were transfected with the dl MMTV IRES plasmid together with a control DNA or plasmids encoding for the wt- hnRNP A1-HA protein, or the mutants hnRNP A1-HA S4A/S6A, hnRNP A1-HA S4D/S6D, hnRNP A1-HA S199A, or hnRNP A1-HA S199D proteins. Expression of the total hnRNP A1 and the HA-tagged proteins were followed by western blotting (Figure 9A). Luciferase activities were measured, and results, presented as RTA, showed that hnRNP A1 mutants hnRNP A1-HA S4A/S6A, hnRNP A1-HA S199A and hnRNP A1-HA S199D significantly (*P* <0.05) enhance MMTV IRES activity to levels similar to that of the wt-hnRNP A1-HA protein (Figure 9B). Strikingly, hnRNP A1-HA S4D/S6D mutant further enhanced MMTV IRES activity ($\sim$94% increase) over the wt-hnRNP A1-HA protein (Figure 9B). These observations indicated that S6K2-induced phosphorylation of hnRNP A1 enables the protein to strongly influenced translation driven by the MMTV IRES.

Then we sought to determine the role of PRMT5-induced SDMA of MMTV IRES activity. HEK 293T cells were transfected with the dl MMTV IRES plasmid, followed by the treatment, or not, with EPZ015666. Western blot analysis confirmed the expression of the endogenous PRMT5 and hnRNP A1, as well as the reduction in SDMA (Figure 9C). RLuc and FLuc activities were determined, and results were expressed as RTA showed that the activity of the MMTV-IRES was significantly (*P* <0.05) increased ($\sim$98–121%) in cells treated with EPZ015666 (Figure 9D). This unexpected observation suggested that PRMT5-induced methylations inhibit MMTV-IRES activity. Intrigued by this observation, we then decided to evaluate the effect of wt-hnRNP A1-HA overexpression when combined with EPZ015666 treatment. For this, HEK 293T cells co-transfected with plasmids dl MMTV IRES and wt-hnRNP A1-HA, were treated, or not, with EPZ015666 (10 μM). Western blot analysis showed that the presence of EPZ015666 reduces SDMA in cells without an evident effect over the expression of the endogenous PRMT5 protein or that of the hnRNP A1 protein (Figure 9E). Interestingly, MMTV-IRES activity was further enhanced ($\sim$52%) when the expression of wt-hnRNP A1-HA was combined with EPZ015666 treatment (Figure 9F). This result suggests that PRMT5-induced methylation of hnRNP A1 reduces the ability of the ITAF to stimulate the activity of the MMTV-IRES.

## DISCUSSION

In this study, we provide evidence showing that hnRNP A1, one of the most abundant nuclear proteins (77), participates in HIV-1 full-length mRNA translation (Figure 1). These findings complement a previous report showing HIV-1 replication induces an increased expression of hnRNP A1 that leads to enhanced activity of HIV-1 IRES-mediated translation initiation (23). Furthermore, we provide new experimental controls to unequivocally demonstrate that hnRNP A1 is an ITAF for the HIV-1 IRES (Figures 2 and 3). We also show that hnRNP A1 is an ITAF for the HTLV-1 and MMTV IRESs (Figures 6 and 8). Interestingly, hnRNP A1 overexpression stimulated the MMTV IRES to a much greater extent ($\sim$13-fold increase) than it did to the HTLV-IRES ($\sim$5-fold increase) or the HIV-1 IRES ($\sim$1.5-fold increase). So, even though hnRNP A1 is an ITAF for these retroviral IRESs in HEK 293T cells, their activities are differentially regulated by the protein. The finding that retroviral IRESs share common ITAFs is not novel as HIV-1, HTLV-1, and MMTV IRESs also require the hypusinated-eIF5A protein for their function (29). Nonetheless, retroviral IRESs do not share all ITAFs. For example, the polypyrimidine tract-binding protein (PTB) binds to the 5′UTR of the MMTV full-length mRNA, promoting MMTV IRES activity (35). However, PTB does not interact with the HIV-1 5′UTR, nor does it regulate HIV-1 IRES activity (35,79). Therefore, these findings confirm that retroviral IRES activity requires ITAFs to be fully functional and suggest that most probably more than one ITAF is needed. Furthermore, they suggest that different retroviral IRESs may share ITAFs, or not. However, shared ITAFs might differentially regulate translation from their different targeted retroviral mRNAs.

In contrast to most viral IRESs that rely mainly on their RNA structure to function (80,81), cap-independent translation initiation of cellular mRNAs requires the assembly of ribonucleoprotein (RNP)-complex, the IRESome (82). Studies of cellular IRESs show that the ITAF composition of the IRESome, as well as the PTMs of these ITAFs, regulate their translational activity. Interestingly, the fine-tuning (increase or decrease) of cellular IRES activity by hnRNP A1 PTMs has been well-documented (40–42,49,50,77). Thus, it was of interest to explore the possible role of hnRNP A1 PTMs on retroviral IRES function. We focused only on hnRNP A1 PTMs known to fine-tune cellular IRES function (Figure 4A). When we evaluated S6K2 and Akt induced hnRNP A1 phosphorylations at sites S4/S6 and S199, respectively, we found that they did not severely impact the ability of the protein to stimulate the HIV-1 IRES (Figure 4B and C) or the HTLV-1 IRES (Figure 7A and B). In contrast, S6K2-induced phosphorylations of hnRNP A1 sharply increase MMTV IRES activity (Figure 9A and B). Thus, S6K2-induced phosphorylations of hnRNP A1 enable the ITAF to promote MMTV IRES activity further when compared to its nonphosphorylated version of the protein (Figure 9B). These results show that when post-translationally modified, the same ITAF might differentially impact on the activity of dissimilar retroviral IRESs. To further this notion, we also evaluated the impact of type II arginine methyltransferase PRMT5 SDMA of hnRNP A1 on retroviral IRES activity. PRMT-5 induced methylation of hnRNP A1 on sites R218/R225 promoted the activity of the HIV-1 (Figure 4D and E) and the HTLV-1 IRES (Figure 7E and F). Sites R218/R225 of hnRNP A1 are located in the proteins Gly-rich-domain that mediates RNA binding and also the cooperative binding of hnRNP A1 with itself and other hnRNPs (41,83). Interestingly, the Gly-rich-domain is not essential for the functions of hnRNP A1 in alternative splicing of the HIV-1 mRNA (75). Consistent with a previous report (41), the loss of PRMT5 activity resulted in a reduction of hnRNP A1 binding to the dl-target RNA (Figure 5F and G). The reduction of hnRNP A1 binding to the HIV-1 IRES containing mRNA correlated with the decrease of IRES activity (Figures 4D, E, and 5). These results indicate that the hnRNP A1 function over the HIV-1 IRES requires direct binding of the protein to the mRNA. However, the methylation of arginine residues also alters the shape of the amino acids side chain as well as its hydrogen bonding patterns modifying protein–protein interactions (84). Thus, at this stage, we cannot entirely discard the possibility that methylations in the Gly-rich domain of hnRNP A1 might also impact the overall protein composition of the HIV-1 RNP-complex that regulates IRES-mediated translation initiation. Further experiments, not included in this study, are required to ascertain or refute this possibility. The reduction of PRMT5-induced SDMA, due to the pharmacological inhibition, induced a decrease, but not a total translational shutdown of HIV-1 IRES-mediated translation initiation, indicating that PRMT5 induced methylation fine-tune the activity of the HIV-1 IRES (Figures 4E and 5). At this stage, we cannot ascertain that the reduction in HIV-1 IRES activity in the presence of EPZ015666 (Figure 5A) is exclusively associated with hnRNP A1. PRMT5 might be simultaneously targeting other ITAFs parts of the RNP-complex associated with HIV-1 IRES activity. Further experiments, not included in this study, are required to address this issue.

In contrast, to the findings with the HIV-1 and HTLV-1 IRESs, the activity of the MMTV IRES was enhanced in the presence of EPZ015666 (Figure 9D). This observation suggested that PRMT5-induced methylation of hnRNP A1 or another unidentified ITAF targeted by the drug downregulates MMTV IRES activity (Figure 9C). This was an unexpected observation given the impact of hnRNP A1 on MMTV IRES activity (Figure 8C and D). Nonetheless, when directly assessed, hnRNP A1 was capable of stimulating the MMTV IRES even in the presence of EPZ015666 (Figure 9E and F). When the drug was present, the ability of hnRNP A1 to stimulate the activity of the MMTV IRES further increased (Figure 9E and F). This observation partially confirmed our conclusion by indicating that PRMT5-induced methylation of hnRNP A1 inhibits the ITAFs ability to stimulate the MMTV IRES. The mechanism by which hnRNP A1 enhances the activity of the MMTV IRES in the presence of EPZ015666 remains unclear. However, our finding suggests that the molecular mechanisms driving MMTV IRES activity in the presence of hnRNP A1 most probably differ from those associated with the HIV-1 and HTLV-1 IRESs. It is tempting to speculate that the differences observed between the HIV-1 and HTLV-1 IRESs, and the MMTV IRES in the presence of EPZ015666 is related to the composition of the different RNP-complexes driving cap-independent translation initiation. Further experiments are required to reveal the biological significance of these observations and to fully understand the molecular mechanism driving the difference in IRES activity between the HIV-1, HTLV-1 and MMTV IRESs. However, this study clearly showed that the same hnRNP A1 PTM could have contrasting effects on different retroviral mRNA targets.

In summary, the results presented in this manuscript show that the translation control exerted by a single ITAF varies substantially amongst several retrovirus IRESs. The results also demonstrate that PTMs of hnRNP A1 by phosphorylation and PRMT5-induced methylation fine-tune retroviral IRES-mediated translation initiation. The work described herein clarifies that additional levels of regulation mediated by cellular proteins and their PTMs are at play in the translational control of retroviral cap-independent translation initiation.

## Supplementary Material

gkaa765_Supplemental_Files
