# Supplementary material for: Post-translational modifications of hnRNP A1 differentially modulate retroviral IRES-mediated translation initiation
Source: Nucleic Acids Res. 2020 Sep 22;48(18):10479–99. doi: 10.1093/nar/gkaa765 (PMC7544202; doi:10.1093/nar/gkaa765)
Supplement: gkaa765_Supplemental_Files [file gkaa765_supplemental_files.zip › Supplemental FIGURE and FIGURE LEGENDS-revised_ALDO.docx]

**Supplemental FIGURE and FIGURE LEGENDS.**

**
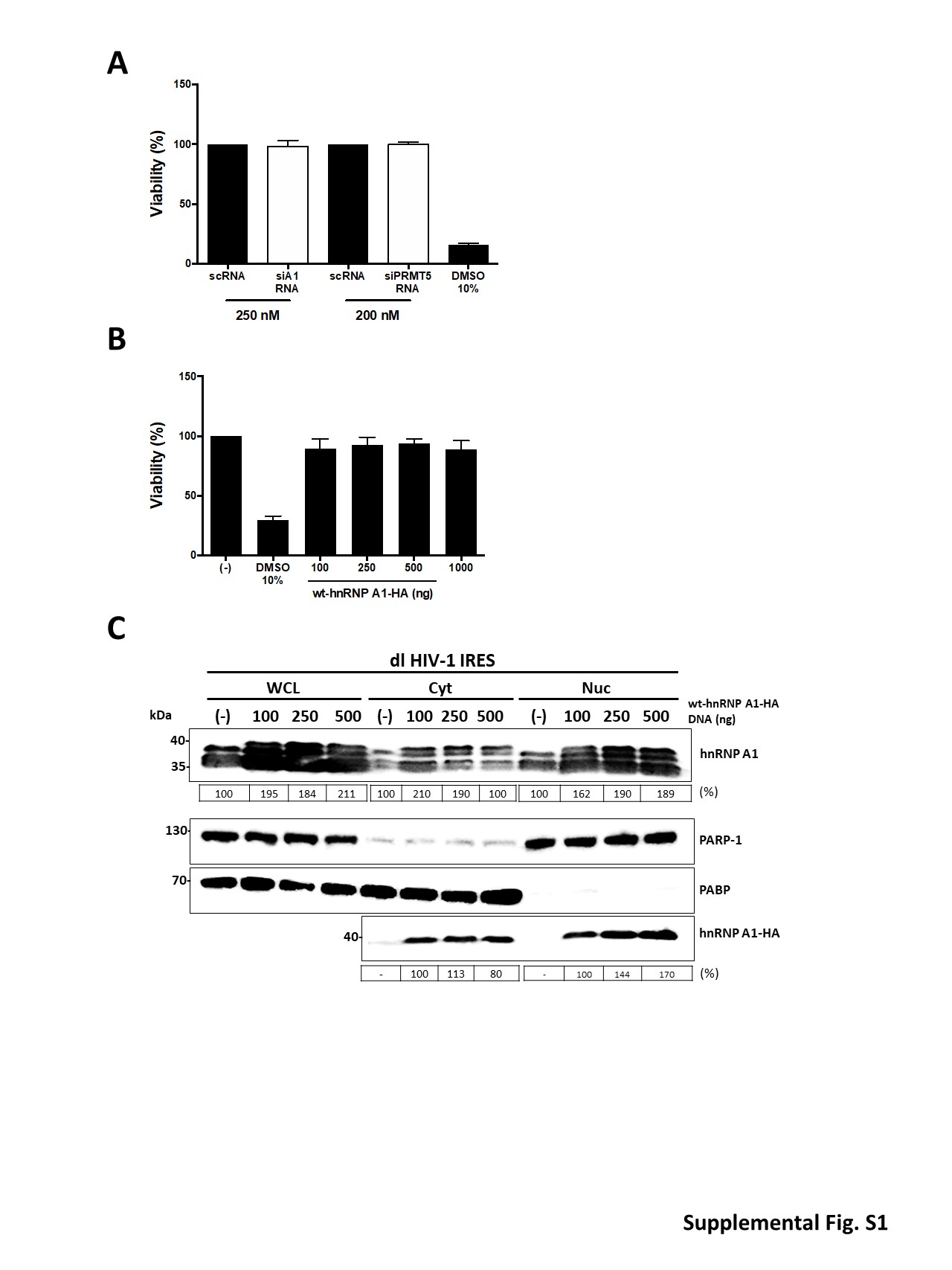
**

**Figure S1. Knockdown and overexpression of hnRNP A1 in HEK 293T cells. (A)** HEK293T cells were transfected with a silencer RNA targeting the hnRNP A1 mRNA (siA1; 250 nM). A scrambled RNA (scRNA; 250 nM) was transfected as a negative control. **(B)** HEK293T cells were transfected with increasing concentrations (100-1000 ng) of the wt-hnRNP A1-HA plasmid. **(A and B)** Cell viability was determined as described in the Materials and Method section using dimethylsulfoxide (DMSO, 10%) as a control for cell death. Data are expressed relative to the viability of cells transfected with scRNA **(A)** or untransfected wt-hnRNP A1-HA **(B)** set to 100%. Values shown are the mean (+/- SEM) for three independent experiments, each performed in duplicate. Statistical analysis was performed by an ordinary one-way ANOVA test (*P < 0.05). (C) HEK 293T cells were transfected with the dl HIV-1 IRES plasmid together with different amounts of wt-hnRNP A1-HA plasmid, and 24 hrs post-transfection (hpt) cells were collected and resuspended in PBS. One-third of this cellular suspension was lysed with 500 µL of RIPA buffer and sonicated (whole cell lysate [WCL]). Two-third were lysed with 500 µL of RLNa buffer, and supernatant (cytoplasmic fraction [Cyt]) was rescued and pellet left (nuclear fraction [Nuc]) was resuspended with 500 µL of RIPA buffer and sonicated. Cellular extracts were used for western blotting using mouse anti-hnRNPA1 in the upper panel. Nuclear and cytoplasm protein markers were detected using mouse anti-PARP-1 and mouse anti-PABP in middle panels, respectively. Mouse anti-HA antibody was used at the bottom panel. The relative levels of hnRNP A1 and hnRNP A1-HA proteins were estimated based on the intensity of immunoreactive bands by image-J, as detailed in Material and Methods.


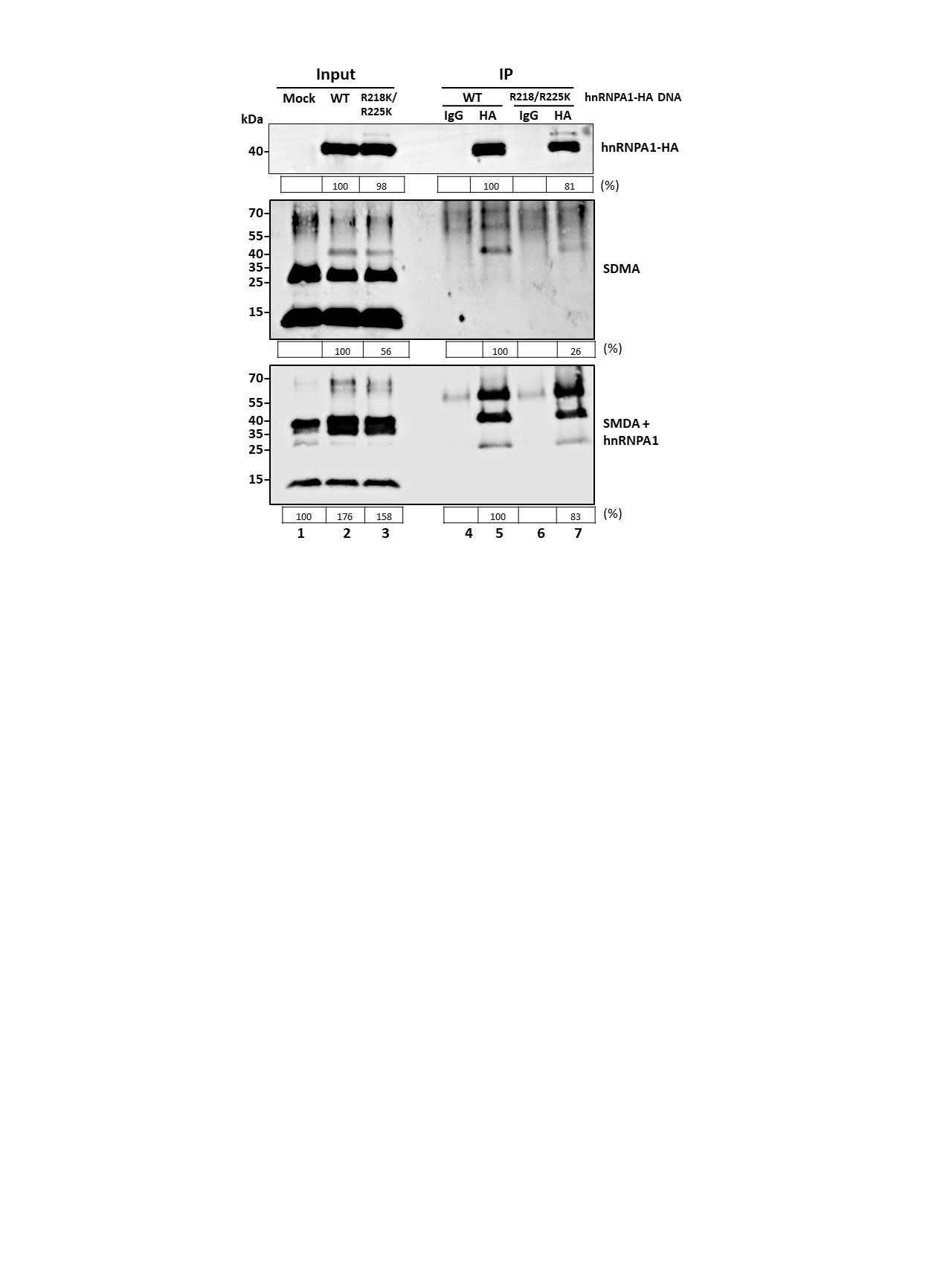


**Figure S2. Symmetric dimethylation of hnRNP A1-HA is impaired in the R218/R255K mutant protein.** HEK 293T cells were transfected with the wt-hnRNPA1-HA or R218/R255K mutant plasmid, and 24 hpt cells were collected and lysed with CoIP buffer. Cellular extracts were used for immunoprecipitation with protein A/G PLUS-Agarose plus mouse anti-HA antibody or mouse IgG control. The beads were washed four times and incubated with loading buffer at 95ºC to rescue the supernatant, which was used for western blotting. The upper panel, a mouse anti-HA, in the middle panel, a rabbit anti-SDMR, in the bottom panel, a mouse anti-hnRNPA1 are used as primary antibodies in the western blots. The recombinant protein A/G conjugated with HRP was used as the secondary antibody in the upper and middle panel, while a goat anti-mouse conjugated with HRP was used as a secondary antibody in the bottom panel. The relative amounts (%) of hnRNP A1-HA protein of the upper panel, and proteins present at ~ 40 kDa of the middle and bottom panels, were estimated by image-J.


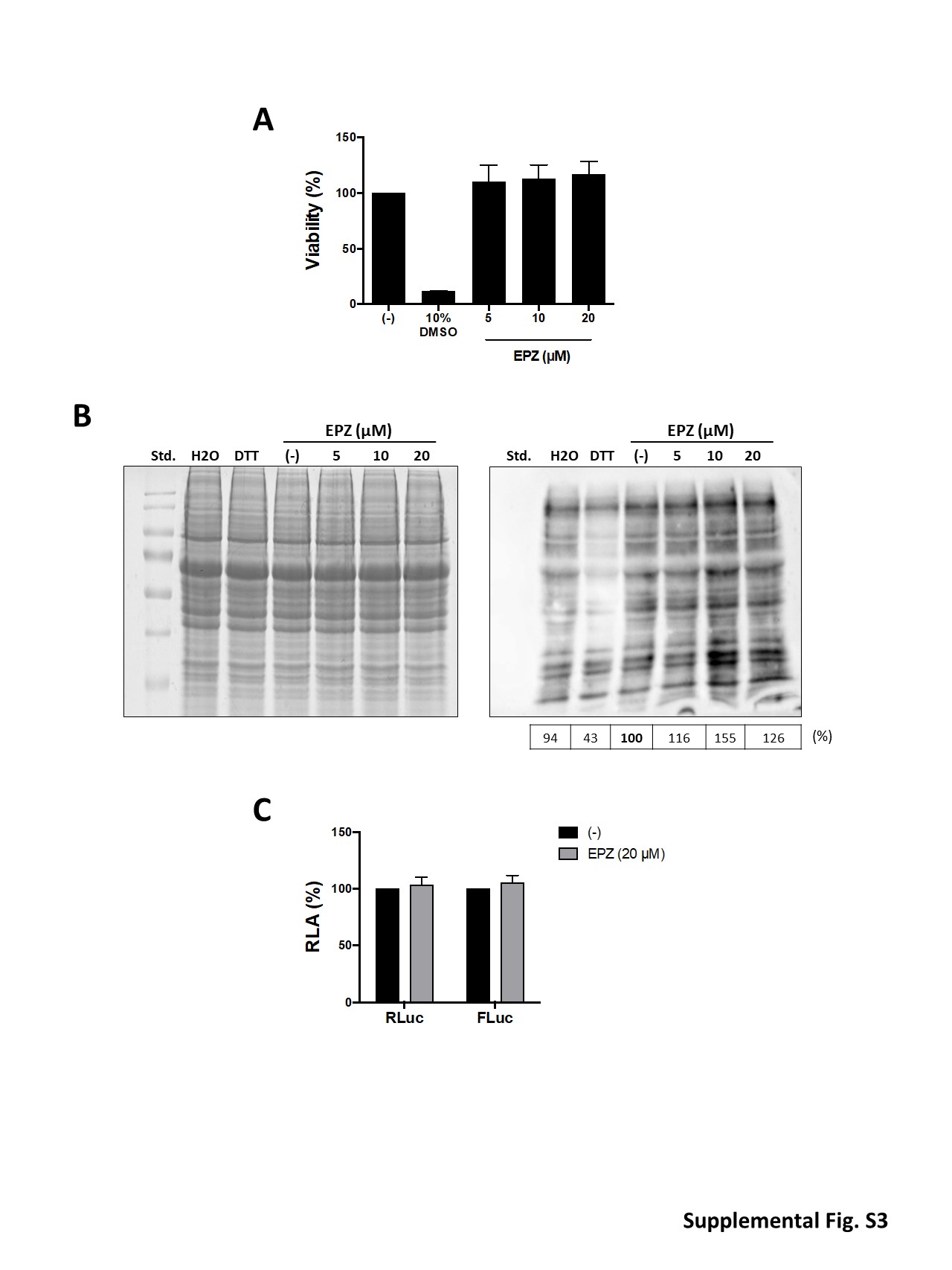


**Figure S3. Impact of EPZ015666 treatment in HEK293T cells. (A)** Cell viability was determined as described in the Materials and Method section. HEK293T cells were treated with increasing concentrations of EPZ015666 (5-20 μM) for 48 h. Dimethylsulfoxide (DMSO, 10%) was used as a control for cell death. Data are expressed in (%) relative to the viability of cells in the presence of the drug vehicle (-) used as a negative control, set to 100%. Statistical analysis was performed by an ordinary one-way ANOVA test. **(B)** The global protein synthesis was determined by the SUnSET method, performed by incubating cells in media supplemented with Puromycin, as indicated in Material and Methods. Western blot was performed to evaluate the amount of Puromycin-labelled proteins in EPZ015666 treated and untreated cells (right panel). The specificity in the anti-puromycin label was determined by including a non-Puromycin treated sample (H_2_O). Dithiothreitol (DTT) was used as a control for the inhibition of protein synthesis. The relative amount of puromycin-labeled protein was estimated by image-J. In parallel, proteins resolved by SDS-polyacrylamide gel electrophoresis (SDS-PAGE, 12%) were stained with Coomassie Blue to verify the equal loading of protein in all lanes (left panel). **(C)** HEK293T were transfected with dl HIV-1 IRES plasmid (200 ng). EPZ015666 (20 µM) was added, or not (-), to cell extracts recovered 24 h post-transfection. RLuc and FLuc activities were measured 30 min post-incubation and expressed as RLA relative to values obtained when only the vehicle (-) was used, set to 100%. Statistical analysis was performed by an ordinary two-way ANOVA test. Values shown are the mean (+/- SEM) for three independent experiments, each performed in duplicate.
